# ITGB1-dependent upregulation of Caveolin-1 switches TGFβ signalling from tumour-suppressive to oncogenic in prostate cancer

**DOI:** 10.1038/s41598-018-20161-2

**Published:** 2018-02-05

**Authors:** Teijo Pellinen, Sami Blom, Sara Sánchez, Katja Välimäki, John-Patrick Mpindi, Hind Azegrouz, Raffaele Strippoli, Raquel Nieto, Mariano Vitón, Irene Palacios, Riku Turkki, Yinhai Wang, Miguel Sánchez-Alvarez, Stig Nordling, Anna Bützow, Tuomas Mirtti, Antti Rannikko, María C. Montoya, Olli Kallioniemi, Miguel A. del Pozo

**Affiliations:** 1 Institute for Molecular Medicine Finland FIMM, University of Helsinki, Biomedicum Helsinki 2U, Tukholmankatu 8, P.O. Box 20, FI-00014, 00290 Helsinki, Finland; 2 Integrin Signalling Lab, Cell Biology & Physiology Program; Cell & Developmental Biology Area, Centro Nacional de Investigaciones Cardiovasculares Carlos III (CNIC), Madrid, Spain; 3 Cellomics Unit, Centro Nacional de Investigaciones Cardiovasculares Carlos III (CNIC, Madrid, Spain; 4 Department of Pathology, Medicum, University of Helsinki, Haartmaninkatu 3 C, 00014 Helsinki, Finland; 5 Department of Pathology, HUSLAB, Helsinki University Hospital, Finland, Haartmaninkatu 3 C, 00029 Helsinki, Finland; 6 Helsinki University and Helsinki University Hospital, Department of Urology, PL340, 00029 HUS Helsinki, Finland; 7 Science for Life Laboratory, Karolinska Institutet, Department of Oncology and Pathology, 171 65 Solna, Sweden; 8 Present Address: Quantitative Biology Department, Discovery Science, AstraZeneca, Unit 310 - Darwin Building, Cambridge Science Park, Milton Road, Cambridge, CB4 0WG UK

## Abstract

Caveolin-1 (CAV1) is over-expressed in prostate cancer (PCa) and is associated with adverse prognosis, but the molecular mechanisms linking CAV1 expression to disease progression are poorly understood. Extensive gene expression correlation analysis, quantitative multiplex imaging of clinical samples, and analysis of the CAV1-dependent transcriptome, supported that CAV1 re-programmes TGFβ signalling from tumour suppressive to oncogenic (i.e. induction of SLUG, PAI-1 and suppression of CDH1, DSP, CDKN1A). Supporting such a role, CAV1 knockdown led to growth arrest and inhibition of cell invasion in prostate cancer cell lines. Rationalized RNAi screening and high-content microscopy in search for CAV1 upstream regulators revealed integrin beta1 (ITGB1) and integrin associated proteins as CAV1 regulators. Our work suggests TGFβ signalling and beta1 integrins as potential therapeutic targets in PCa over-expressing CAV1, and contributes to better understand the paradoxical dual role of TGFβ in tumour biology.

## Introduction

Epithelial-to-mesenchymal transition (EMT) is associated with the disruption of the normal structure of the epithelium and the invasion of carcinoma cells into the surrounding stroma^1^. EMT-associated epithelial plasticity is a relevant phenomenon in prostate cancer (PCa) progression^2^. A hallmark of EMT is the decreased or aberrant expression of the adherens junction protein E-cadherin^3^. In PCa, decreased E-cadherin expression has been shown to correlate with clinical disease progression in multiple independent studies^4–8^. A pivotal regulator of EMT is transforming growth factor beta (TGFβ)^9^. Shutdown of canonical TGFβ signalling through down-regulation or deletion of the transcriptional effector protein, SMAD4, promotes cell growth and proliferation in epithelium, and can therefore lead to carcinogenesis^10^. However, in advanced stages of cancer, the growth-suppressive function of TGFβ is often subverted to promote invasion and EMT, independent of SMAD proteins^11^. Indeed, there is evidence that TGFβ expression correlates with PCa progression and poor clinical outcome^12,13^. Also, TGFβ signalling promotes invasive growth and metastasis of PCa^14–17^. However, and despite its outstanding relevance, the principles determining this “Janus” behaviour of the TGFβ pathway have not been fully elucidated.

Caveolin-1 (CAV1) is a cholesterol-binding scaffold protein which functions in membrane dynamics, uptake of certain viruses, lipid metabolism, signalling, mechano-sensing and membrane mechano-protection^18^. CAV1 is known to homo-oligomerize, hetero-oligomerize with CAV2, and assemble with cavins (1–4) to form membrane invaginations called *caveolae*. CAV3 is the muscle-specific caveolin, and the three isoforms are typically expressed in tissues subjected to wide variations in mechanical stress (endothelium, muscle, lung, fat, etc.). CAV1 positively regulates integrin signalling to promote cell growth through the RAS-ERK pathway^19^. Conversely, integrin-mediated cell adhesion may dramatically affect the localization of CAV1^20^. However, how CAV1 expression is regulated is poorly understood. Moreover, CAV1 interacts with numerous other signalling proteins and pathways, but its impact is highly contextual and tissue-specific. This conditional functionality is also reflected in its suggested roles in cancer, both as a potential suppressor in early disease stages and a proto-oncogene in later stages^21^. The tumour-promoting role of CAV1 is associated both with resistance to apoptosis^22,23^ and increased cancer cell migration or invasion^24^. CAV1 knock-down inhibits migration of PCa cell lines *in vitro*^25^ and reduces lymph node and lung metastasis in experimental metastasis assays^26^, suggesting an active role for CAV1 in PCa progression. In addition, CAV1 may either promote or inhibit EMT depending on tissue context^27–30^. Previous work in PCa suggests that CAV1 induces cell invasion and inhibits E-cadherin expression, which are important hallmarks of EMT^31^. Furthermore, CAV1 overexpression is associated with aggressive disease and poor clinical outcome^32,33^. However, the molecular pathways promoting CAV1 overexpression – linked to increased invasion and potential resistance to apoptosis – are still largely unknown in PCa.

Here, we show that CAV1 expression associates with oncogenic EMT markers and inversely correlates with E-cadherin expression in clinical PCa tissue samples. In addition, we found that CAV1 promotes the expression of oncogenic TGFβ targets, but reduces the expression of TGFβ-induced p21 growth inhibitory protein in PCa cells. Rationally-targeted functional image-based RNAi screenings revealed that CAV1 expression is controlled by beta1 integrins and integrin-related signalling. Multiplexed immunohistochemistry (IHC) and image analysis revealed a high degree of co-expression between beta1 integrin (ITGA2/B1) and CAV1 both in cell lines *in vitro* and in clinical PCa tissue. Our findings suggest that increased CAV1 levels are not merely a consequence, but an active driving element of PCa towards a more mesenchymal phenotype.

## Results

### CAV1 expression associates with a mesenchymal gene signature

**Figure 1:**
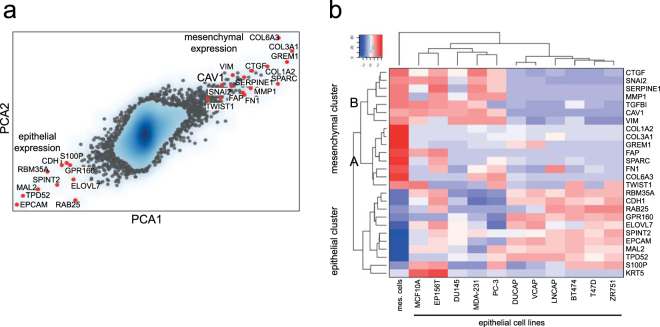
CAV1 associates with EMT gene expression signatures. (**a**) PCA analysis across extensive cancer cell line datasets classifies CAV1 as a robust component of mesenchymal gene expression signature. (**b**) Hierarchical clustering across selected epithelial and mesenchymal cell lines of the highest scoring differentially expressed genes defines a subset of genes described as “transitional EMT”, which includes CAV1 (cluster B).

CAV1 expression has been shown to be up-regulated in PCa and to associate with poor prognosis^32–34^. Nevertheless, despite these reported links, the underlying mechanisms by which CAV1 dysregulated expression determine an aggressive phenotype in PCa are currently not well understood. To gain further insight into the functional programs associated with CAV1 expression in PCa, we queried extensive transcriptome datasets to find signatures exhibiting correlation with CAV1 expression (see Methods). Gene subsets were classified into epithelial and mesenchymal signatures across different epithelial and mesenchymal cell lines using a principal component analysis (PCA) (Fig. 1a; see Supplementary Table S1 for cell lines). We found that the expression of CAV1 was much higher in mesenchymal than in epithelial cells, and CAV1 was among the top 100 ‘mesenchymal-specific’ genes (see Supplementary Table S2 and S3 for top 100 mesenchymal and top 100 epithelial genes, respectively). In a panel of well-characterized prostate and breast cancer cell lines, hierarchical clustering of the top 100 mesenchymal and the top 100 epithelial genes revealed two mesenchymal sub-clusters ‘A’ and ‘B’ (Fig. 1b), defined as “pure” and “transition” clusters, respectively. The genes of the “pure” mesenchymal cluster were exclusively up-regulated in mesenchymal cells, such as SPARC and a few ECM structural proteins, whereas the genes in the “transition cluster” were not only highly expressed across mesenchymal cell lines but also substantially expressed in a number of epithelial cell lines. This “transition cluster”, which comprised six genes (e.g. SERPINE1 (PAI-1), SNAI2 (SLUG), MMP1, VIM (Vimentin), TGFBI, and CTGF)) tightly correlated with CAV1 expression across all cell lines surveyed. Thus, these orthogonal datasets potentially dissect gene subsets specific for EMT expression signature, and support that CAV1 could actively contribute to the regulatory programs driving EMT in PCa.

**Figure 2:**
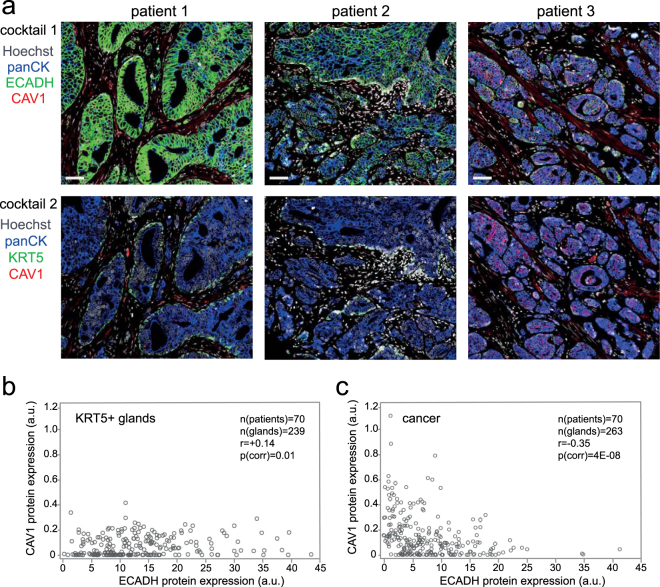
Patients with moderate-to-high CAV1 expression have a partial loss of E-cadherin. (**a**) Two different antibody cocktails were applied to get information on CAV1 and E-cadherin (ECADH) expression in PCa epithelium. Pan-cytokeratin (panCK) was used as a marker for epithelium, cytokeratin-5 (KRT5) for basal epithelium, and Hoechst for nuclei. Example images of patients 1, 2, and 3 showing strong, medium, and weak E-cadherin expression, respectively, with strong CAV1 expression only in the patient 3 (scale bar = 50 µm). (**b**,**c**) Digital analysis of CAV1 and ECADH expression in PCa patients (n = 70) within KRT5-positive or KRT5-negative epithelial areas. r = pearson correlation. Arbitrary unit (a.u.) here is defined as the average protein expression intensity measured: total channel intensity (1–255) divided by total area in pixels.

As the aberrant expression of E-cadherin is a hallmark of EMT and is associated with poor clinical outcome in PCa^6^, we set out to study the association of CAV1 and E-cadherin expression in human PCa tumours. Briefly, we applied fluorescence-based multiplex immunohistochemistry^35^, which allowed both visual scoring and digital quantitative image analysis. Importantly, the method allowed digital segmentation of the epithelium from stroma using pan-cytokeratin (panCK) as epithelial marker. We observed a strong association between CAV1 expression and reduced E-cadherin expression in cancer epithelia (panCK+/KRT5−), but not in KRT5-positive regions, which consist mainly of non-malignant epithelium (benign, PIN), but may also contain malignant tissue (intraductal carcinoma of the prostate, IDCP) (Fig. 2a). This effect was evident not only in sections across different cancer patients (inter-tumour association), but also across different areas within the same patient sample (intra-tumour association) (Supplementary Fig. S1a–b). To further validate these observations, we visually analysed a set of PCa patient samples (TMA_2; n = 70) for CAV1 (visual scores 0–3) and E-cadherin (visual scores 0–5). Patients with high CAV1 expression showed lower E-cadherin expression (corr r = −0.60; p = 4E-8; n = 70). In addition, we digitized the sections and performed automatic image analysis of CAV1 and E-cadherin expression in KRT5-positive glands (n = 239) and in cancerous areas (panCK+/KRT5−) (n = 263). Consistent with visual scoring, we found a significant inverse correlation of CAV1 and E-cadherin in cancerous areas (Pearson corr r = −0.35; p = 4E-8), whereas a weak positive correlation was found in KRT5-positive glands (Pearson corr r = + 0.14; p = 0.01) (Fig. 2b,c). Furthermore, we found that CAV1 expression was generally higher (p = 1.8e-13) and E-cadherin expression lower (p = 8.4e-12) in cancerous epithelium as compared to KRT5-positive epithelium. Analysis of mRNA expression data from the GeneSapiens database^36^ recapitulated the inverse correlation of *CAV1* and *CDH1* (E-cadherin) specifically in clinical PCa samples (correlation coefficient = −0.19; p < 0.001; n = 460), but not in the benign prostatic tissue (correlation coefficient = 0.034; p = 0.63; n = 208) (Supplementary Fig. S1c). In accordance with our correlation analysis in cell lines (see Fig. 1), the inverse correlation between CAV1 and E-cadherin expression in primary PCa epithelium prompted a model whereby CAV1 actively participates in the rewiring of cell signalling towards EMT during PCa progression.

### CAV1 polarizes the reprogramming of TGFβ-associated transcriptomic signatures

**Figure 3:**
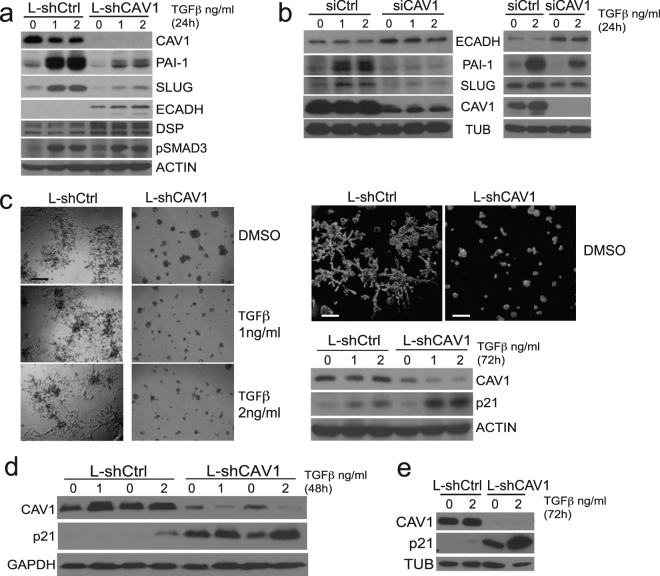
CAV1 promotes oncogenic TGFβ signalling. (**a**) PC-3 cells silenced and treated with TGFβ as indicated. pSMAD3 was used as a positive control for TGFβ induction. (DSP = Desmoplakin). (**b**) Same as (**a**), except cells were DU145 (left panel) or PNT2 (right panel). (**c**) PC-3 cells were grown on top of Matrigel (50%), silenced as indicated, and treated with TGFβ for 72 h. Phase contrast bright-field (left panel) and high magnification dark-field images (upper right panel), with indicated targets blotted from a replicate experiment (lower right panel). Scale bars 300 µm (left) and 30 µm (right). (**d**) PC-3 cells were cultured in suspension, silenced as indicated, and treated with TGFβ. (**e**) Same as (**d**), except cells were grown inside collagen type I.

Because TGFβ is one of the most potent inducers of EMT and a well-established regulator of E-cadherin expression^2^, we decided to explore TGFβ-associated regulatory events upon CAV1 silencing in PCa cells. We performed a genome-scale microarray analysis of PC-3 cells stably expressing CAV1-targeting shRNA (L-shCAV1) or non-targeting shRNA (L-shCTR) as control. Intriguingly, CAV1 silencing differentially affected distinct subsets of *bona fide* TGFβ targets: 24 genes were down-regulated and 22 genes were up-regulated (Supplementary Table S4). Moreover, CAV1 silencing reduced the expression of TGFβ target genes in the mesenchymal gene cluster but increased those in the epithelial gene cluster (Supplementary Tables S4 and S5). Functional network analysis of the differentially regulated genes recapitulated the bimodal segregation of TGFβ targets in two modules – first predominantly comprised of genes down-regulated upon CAV1 silencing (“ECM remodelling-invasion”) and the second composed of genes showing inverse correlation with CAV1 (“Inflammation”) (Supplementary Fig. S2). Interestingly, both modules were enriched in distinct genes for potential upstream regulatory kinases, further suggesting a functional coherence of the genes within these modules (Supplementary Fig. S2). We confirmed the expression of a subset of these genes by Western blotting or qPCR, followed by a validation upon CAV1 knockdown with an independent CAV1 siRNA duplex (Fig. 3a; Supplementary Fig. S3a,b). CAV1 silencing in three prostate cell lines reduced the expression of two well-characterized mesenchymal TGFβ targets, SLUG (SNAI2) and PAI-1 (SERPINE1) (Fig. 3a,b; Supplementary Fig. S3a,b, S3d,e), but increased the expression of TGFβ targets that are known positive regulators of epithelial integrity (DSP = desmoplakin; ITGB4 = integrin beta4; CDH1 = E-cadherin) (Fig. 3a; Supplementary Fig. S3b; Supplementary Table S4). Critically, the suppression of CAV1 expression did not alter SMAD3 phosphorylation, nor affected the subcellular localization of either SMAD2 or SMAD3 (Fig. 3a; Supplementary Fig. S3b,c), supporting that the observed effects are not likely a consequence of primary disruption of, or insensitivity to TGFβ stimulation, but rather a result of selective rewiring of networks downstream from CAV1.

Because CAV1 silencing affected the phenotype of PC-3 cells cultured on Matrigel (Fig. 3c), we inquired whether CAV1 could also regulate the levels of the TGFβ-induced growth inhibitor, p21 (CDKN1A). First, we found that p21 expression was increased upon TGFβ stimulation in PC-3 cells in three culture conditions: on top of Matrigel (Fig. 3c), in suspension culture (Fig. 3d), and inside collagen type I (Fig. 3e). However, we also observed that CAV1 silencing alone increased p21 expression, mimicking the effect of TGFβ. Importantly, CAV1 silencing synergized with TGFβ stimulation leading to even higher p21 expression.

### CAV1 promotes mesenchymal phenotype in prostate cancer cells

**Figure 4:**
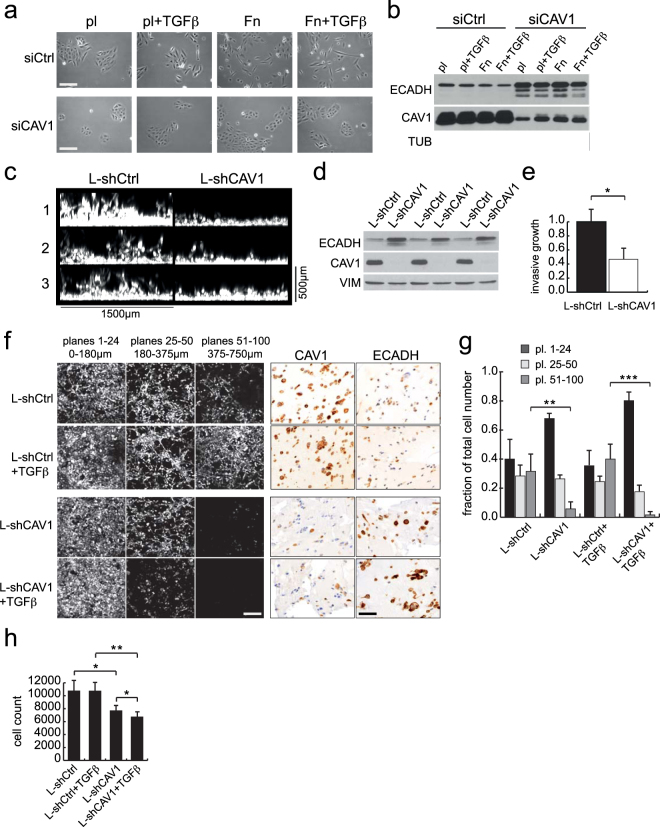
Caveolin-1 promotes EMT and invasiveness, but reduces TGFβ-induced growth inhibition. (**a**) Phase contrast images of RNAi-treated DU145 cells on plastic (pl) or fibronectin-coated cell culture plates (Fn) induced with TGFβ (1 ng/ml) for 70 hours (size bar = 50 µm). (**b**) Same as (a), but Western blot analysis after 72 hours. (**c**) Representative images of side projection views of invasion assays in 50% Matrigel towards serum gradient. PC-3 cells were silenced as indicated and stained with fluorescent phalloidin and Hoechst 33342 and imaged in x-y-z dimensions using confocal microscope. L-shCtrl and L-shCAV1 state for Lentiviral constructs for control-silencing and CAV1-silencing, respectively. (**d**) A replicate invasion experiment of (c), but now cells were analysed using Western blotting. (**e**) Quantification of invasive growth from three independent experiments. The results show fold change of growth area measured. *p < 0.05; two-tailed *t* test. (**f**) Same as (**c**), but now cells cultured in collagen type I (1 mg/ml) with or without TGFβ. The images are maximum projections from consecutive z-planes as indicated. The silencing of CAV1 and subsequent up-regulation of ECADH was evaluated from parallel 3D experiments, which were processed for fixation and parafinization for immunocytochemistry (right panels) (scale bar = 50 µm). (**g**) Relative end-point cell counts in collagen invasion assays from (**f**) in different planes as indicated. Results are from three independent experiments (pl. = confocal planes). (**h**) 3D cell growth measured as total cell count in all the confocal planes. n = 3 independent experiments; *p < 0.05; **p < 0.01; ***p < 0.001; two-tailed *t* test. Error bars represent mean standard deviation.

As CAV1 knock-down influenced EMT-associated signalling in PCa, we decided to explore the effects of CAV1-silencing on cell motility and invasion phenotypes in PCa cell lines. First, DU145 cells were cultured in the presence or absence of TGFβ on two different substrates (plastic vs. fibronectin (FN) coated plates). In agreement with the suggested proactive role of CAV1 in EMT, CAV1 silencing led to reduced scattering and to increased E-cadherin expression upon exposure to FN and/or TGFβ as compared to wild-type control (Fig. 4a,b). Further, CAV1 depletion inhibited invasion in a 3D cell culture model of highly invasive PC-3 cell line (Fig. 4c–e), and this effect was evident either in the presence or absence of TGFβ (Fig. 4f,g). In agreement with our previous analyses of primary tumours, immunocytochemistry of a 3D-cell culture model upon formalin-fixation and paraffin-embedding (FFPE) recapitulated the inverse correlation in CAV1 and E-cadherin protein expression (Fig. 4f, right panel). In addition, CAV1 knock-down significantly suppressed 3D cell growth, which was further inhibited by the addition of TGFβ (Fig. 4h). These results highlight the role of CAV1 in promoting EMT and invasive growth of PCa cells.

### Targeted image-based screens across CAV1 co-expressing transcriptome subsets reveal novel beta1 integrin-dependent modules driving CAV1 expression

**Figure 5:**
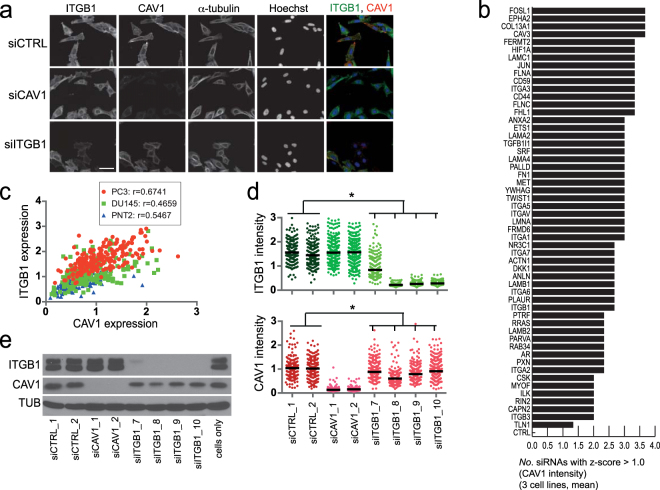
ITGB1 is an upstream regulator of CAV1 expression. (**a**) Example images of the RNAi screen read-outs of PC-3 cells silenced as indicated (size bar = 10 µm). (**b**) Positive regulators of CAV1 expression indicated as number of siRNAs with average z-score higher than 1.0 in three cell lines (PC-3, PNT2, DU145). Each z-score is a mean of three replicate experiments. (**c**) Correlation of CAV1 and ITGB1 expression in three prostate cell lines. Data was extracted from the screen results using siCTRL wells from one single replicate plate. r = pearson correlation. (**d**) Silencing effects of indicated siRNAs on ITGB1 (upper panel) and CAV1 (lower panel) expression at single-cell level in PC-3 cell line from one replicate experiment. Each dot represents expression of ITGB1 or CAV1 in a single cell. Median values are indicated as bars. *p < 0.05; two-tailed *t* test. Each ITGB1 siRNA effect was compared with the mean result of the two siCTRLs. (**e**) Same as in (**d**), but now an independent experiment with cellular lysates analysed using WB.

Given that CAV1 promoted invasive phenotypes and was found to be over-expressed in a subset of PCa patients, characterization of the elements and architecture of the upstream signalling controlling CAV1 levels is warranted. To attain increased sensitivity for high-confidence genes and functional pathways participating in CAV1-associated phenotypes, we first performed an independent, CAV1-centered co-expression profiling across transcriptomic data on 300 different cell lines derived from 25 different tissue types^37^. Top 500 genes correlating with CAV1 expression were queried for annotated functional categories (David Gene Functional Classification tool; http://david.abcc.ncifcrf.gov; ref.^38^). Top GoTerm categories included cell adhesion (p = 6.9E-16), cell-substrate junction (p = 4.1E-16), and cytoskeletal protein binding (p = 4.2E-14) (Supplementary Table S6), which reflect functional annotations for CAV1. We curated these highly co-expressed gene sets as well as functionally-related genes and performed a targeted image-based RNAi screen in three different prostatic cell lines to detect the effect of specific gene-silencing on CAV1 (Supplementary Table S7 for gene list; Supplementary Table S8 for siRNAs). In addition to CAV1, we also quantified the expression of integrin beta1 (ITGB1) (Supplementary Fig. S4a for z-prime values), because it is known to physically interact with CAV1^39^ but also because ‘cell adhesion’ scored as the functional category correlating strongest with CAV1 expression. Examples of representative images and scatter plots for CAV1 and ITGB1 staining intensities across the tested conditions are shown in Figure 5a and Supplementary Figure S4b, respectively. The screen results are summarized in Figure 5b. The screen revealed a highly significant correlation (r = 0.47–0.67; p < 0.01) between ITGB1 and CAV1 expression in all three prostate cell lines studied (Fig. 5c). Mechanistically, all four ITGB1 siRNAs resulted in a pronounced down-regulation of CAV1 protein expression in PC-3 cells – but not vice versa, supporting a specific hierarchical dependency of CAV1 expression on ITGB1 levels (Fig. 5d). The result was further validated by Western blot analysis (Fig. 5e), and also by showing that cell adhesion to integrin beta1 substrates, fibronectin or Matrigel, induces CAV1 expression (Supplementary Fig. S4c). The finding that various ITGB1 binding alpha subunits (ITGA1, ITGA2, ITGA3, ITGA5, ITGA6) and extracellular matrix (ECM) proteins that bind integrins (fibronectin, laminins and collagens), as well as proteins linking integrins with the cytoskeleton (e.g. FERMT2/Kindlin-2, PXN/paxillin, ACTN1/alpha-actinin, ILK/integrin-linked kinase), were identified as positive CAV1 regulators (Fig. 5b), provides further evidence for the role of beta1 integrin as a CAV1 upstream regulator.

### CAV1 over-expression is strongly associated with ITGB1 expression in PCa clinical samples

**Figure 6:**
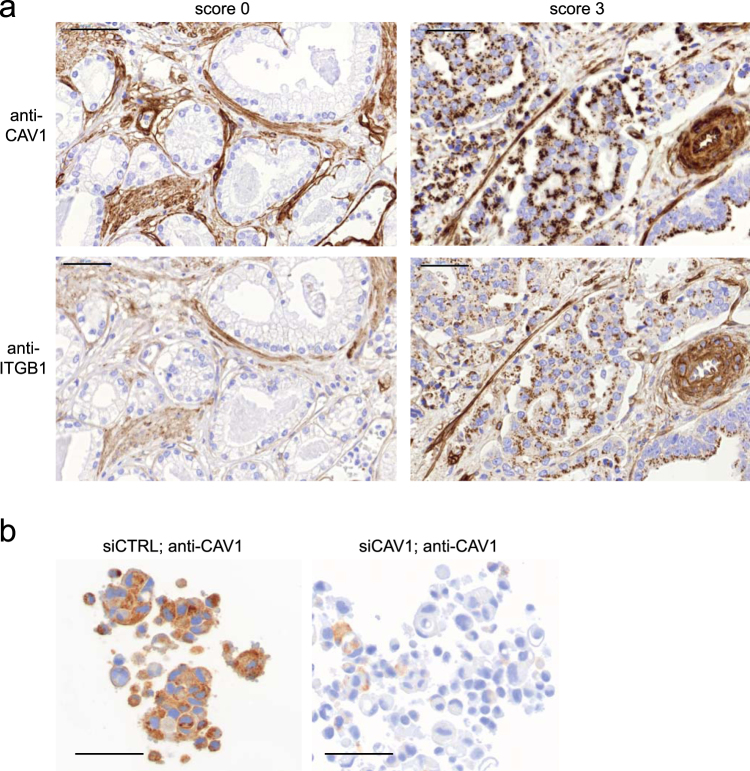
CAV1 overexpression associates with ITGB1 expression in clinical PCa. (**a**) Example images of CAV1 antibody (BD clone 2297; 1/1000) and ITGB1 antibody (EP1041Y; 1/500) IHC of patient cancer cores in TMA_1 cohort with expression scores 0 and 3. (**b**) A control experiment showing high specificity of CAV1 antibody (clone 2297). Silencing of CAV1 in PC-3 cell line substantially decreases the CAV1 expression in IHC staining of paraffin-embedded sample section processed similarly as the tissue sections in the TMA cohort for CAV1 staining (TMA_1) (scale bar = 50 µm).

As we showed that ITGB1 is an upstream regulator of CAV1 expression and the two proteins are correlated *in vitro*, we asked if this correlation could be validated in a large clinical prostate cancer cohort. For this, CAV1 and ITGB1 were analysed in a cohort of 435 patients (TMA_1) in a tissue microarray (TMA) format, including one core from a benign area and three cores from cancer areas in each patient. CAV1 expression in cancer was strong in 11.5% (CAV1 average score x ≥ 2), moderate in 14% (score 1 < x < 2), weak in 48% (score 0 < x ≤ 1), and negative in 27% (score x = 0) of the patients (Table 1). Most of the patients (91%) showed either weak (42%) or negative (49%) CAV1 expression in the benign luminal epithelium. In accordance with previous studies, the mean expression of CAV1 was 71% higher in cancer compared to the benign tissue (p < 0.001, Welch’s one-way ANOVA). Importantly, also the mean expression of ITGB1 was higher in the cancer areas (215%; p < 0.001, Welch´s one-way ANOVA). Representative images of cancer areas with different expression scores for CAV1 and ITGB1 as well as a control experiment for CAV1 antibody IHC validation are shown in Figure 6a and b, respectively. CAV1 and ITGB1 expression strongly correlated across all tissue cores (Spearman rho = 0.492; p < 0.001). Neither CAV1 nor ITGB1 expression associated with Gleason score or pathological tumour stage (pT) (Tables 2 and 3). To support these data, a multiplexed 4-color IHC and automatic image analysis demonstrated that epithelial CAV1 correlates not only with ITGB1 but also with ITGA2 and ITGA6 – alpha integrin subunits forming dimers with ITGB1 (Supplementary Fig. S5).

**Table 1:** CAV1 expression distribution in benign and cancer tissue areas in TMA_1.

CAV1 score	Benign, n (%)	Cancer, n (%)	p-value^1^
Negative (x = 0)	215 (49.4)	117 (26.9)	<0.001
Weak (0 < x ≤ 1)	183 (42.1)	207 (47.6)	
Moderate (1 < x < 2)	0 (0)	61 (14.0)	
Strong (2 ≥ x)	3 (0.69)	50 (11.5)	
Total	401 (100)	435 (100)	

^1^The difference of CAV1 expression between benign and cancer areas was analysed using oneway ANOVA.

**Table 2:** CAV1 expression association with pathological variables in TMA_1.

Gleason score	n (%)	CAV1 negative	CAV1 weak	CAV1 moderate	CAV1 strong	χ^2^(p-value)
≤6	80 (24)	12	45	13	10	0.075
7	200 (59)	57	90	36	17	
8≤	57 (17)	21	23	6	7	
Total^1^	337 (100)	90	158	55	34	
**Pathological stage**	**n (%)**					0.154
≤pT2	182 (58)	41	89	35	17	
pT3≤	129 (42)	40	57	16	16	
Total^2^	311 (100)	81	146	51	33	

^1^Patients who received neo-adjuvant therapy or were missing Gleason score were removed from the analysis.

^2^Patients who received neo-adjuvant therapy or were missing stage information were removed from the analysis.

**Table 3:** ITGB1 expression association with pathological variables in TMA_1.

Gleason score	n (%)	ITGB1 negative	ITGB1 weak	ITGB1 moderate	ITGB1 strong	χ^2^(p-value)
≤6	83 (25)	22	46	11	4	0.331
7	198 (58)	54	114	23	7	
8≤	57 (17)	25	25	5	2	
Total^1^	338 (100)	101	185	39	13	
**Pathological stage**	**n (%)**					0.698
≤pT2	184 (59)	55	101	19	8	
pT3≤	128 (61)	35	71	18	4	
Total^2^	312 (100)	91	172	37	12	

^1^Patients who received neo-adjuvant therapy or were missing Gleason score were removed from the analysis.

^2^Patients who received neo-adjuvant therapy or were missing stage information were removed from the analysis.

## Discussion

In this study, we contribute novel insights onto how CAV1 promotes EMT and invasion in PCa. We show that CAV1 is up-regulated in tumour epithelium in a subset of PCa patients, and this up-regulation is strongly associated with a significant loss of E-cadherin, a hallmark of mesenchymal conversion. In agreement with such a functional role for CAV1 in PCa, knockdown of CAV1 abolished EMT phenotypic traits and invasiveness in PCa cells. Molecular profiling and unbiased analysis of extensive datasets revealed that CAV1 selectively promotes a pro-oncogenic program from TGFβ signalling, while attenuating subsets of TGFβ targets established as tumour suppressors. Finally, we show that beta1 integrins positively regulate CAV1 expression *in vitro –* an observation that is consistent with a strong positive correlation between beta1 integrin and CAV1 expression in prostatectomy patient cohort using both conventional IHC and multiplexed quantitative fluorescence analysis.

A central observation in our study was that CAV1 specifically promotes the expression of oncogenic TGFβ targets including SLUG and PAI-1, apart from down-regulating E-cadherin and inhibiting the basal and TGFβ-induced expression of the cyclin-dependent kinase inhibitor, p21. Previously, SLUG was shown to promote TGFβ-mediated EMT and invasion in PCa cell lines^40–44^, whereas PAI-1 was essential for TGFβ and EGF mediated cell scattering in an EMT model of transformed human keratinocytes^45^. Supporting our findings, Liang and others^27^ showed that CAV1 induces the expression of SLUG and inhibits that of E-cadherin in bladder cancer cell lines. While no previous reports elucidate the molecular pathways explaining the tumour suppressive-to-oncogenic switching of TGFβ signalling in PCa, other studies in different cancers point-out RAS-MEK-MAPK signalling as an important escape mechanism from the growth inhibitory effects of TGFβ^46–48^. Our findings demonstrate that CAV1 promotes invasive growth and inhibits the growth inhibitory effects of TGFβ, as well as reveal the specific suppressive and oncogenic transcriptional targets associated with TGFβ stimulation.

We have previously shown, as opposed to PCa cells, that CAV1 deficiency induces EMT in primary mesothelial cells during peritoneal dialysis (PD)^49^. This apparent contradiction may derive from cell-specificity and from the differences in the tissue microenvironment, as in PD the peritoneum is exposed to pro-inflammatory and pro-fibrotic stimuli. Strippoli and others^49^ showed that CAV1 inhibits the RAS-MEK-ERK activity in primary mesothelial cells thus contributing to inhibition of EMT by this pathway. In the cancers of prostate, colon, lung, melanoma, and Ewing sarcoma, CAV1 has been shown to activate the RAS-ERK-MEK signalling and promote cancer progression^50–54^, demonstrating that in certain cancer tissue the role of CAV1 in this pathway is opposite as compared to mesothelial cells. Likewise, in breast cancer, where CAV1 role has been suggested tumour suppressive, CAV1 inhibits the MEK-ERK signalling^55^, and suppressive role of CAV1 may be observed in pancreatic and ovarian tumours, where CAV1 maintains E-cadherin expression stabilizing cell junctions^30,56^. This kind of cell-specific context-dependency is also reflected in the present study, where we show that CAV1 expression has strong inverse correlation with E-cadherin in KRT5-negative tumour cells, but weak positive correlation in KRT5-positive prostatic glands, which are mostly benign (see Figure 2).

In this study, we also investigated the upstream regulators of CAV1 in prostate cell lines by RNAi and single cell quantitative imaging and found that beta1 integrins positively regulate CAV1 expression. The presence of beta1-interacting alpha-integrins (ITGA2, ITGA5, ITGA6), multiple integrin–actin linker proteins (e.g. PXN, ACTN1, PARVA, FLNA/C, PALLD), as well as signalling adaptors for integrins (e.g. ILK, PLAUR, CD44) as hits in the siRNA screen, further validate beta1 integrin regulation of CAV1 expression. We also show significant co-expression of CAV1 and beta1 integrin proteins in prostate cell lines and translate this finding also to patient tissue samples using both conventional IHC and multiplexed immunostainings with digital image analysis. CAV1 over-expression has been earlier associated with higher Gleason grade and progression of PCa^32–34^. We, however, could not show any association of CAV1 overexpression with either Gleason grade or pathological tumour stage. The reasons for this inconsistency could be due to a different antibody used. We used in this study a monoclonal antibody from BD (6100407; clone 2297), when Yang *et al*.^33^ and Karam *et al*.^32^ performed IHC with a rabbit polyclonal antibody from Santa-Cruz (antibody IDs not stated), and Moon *et al*.^34^ with a rabbit polyclonal antibody from BD (610059).

Do our *in vitro* observations translate to *in vivo* biology? PC3 and DU145 cell lines are derived from distant metastases, whereas PNT2 is an SV40-immortalized “normal” prostate cell line^57–59^. These cell lines do express CAV1, but for example exhibit poor expression levels of the androgen receptor – often reported as key for the primary PCa and metastatic progression^60^. Also, the fact that PNT2 cells exhibit low invasiveness^61^ suggests that CAV1 is necessary but not sufficient per se to drive invasion. However, our results demonstrate that CAV1 upstream regulation is shared by these cell lines and that signalling programmes downstream of TGFβ are specifically impacted by CAV1 downregulation across the models. As the analysis of patient material shows a very strong association of high CAV1 expression with EMT hallmarks (i.e. downregulation of E-cadherin) as well as with the expression of beta1-integrins, we conclude that our *in vitro* models reflect the human *in vivo* biology for this part.

Overall, our results warrant for further parallel assessment of the expression of CAV1 and different integrins as well as the related genes identified in this work, to study whether a positive association could reflect clinical outcomes, and whether these signatures could be diagnostic/prognostic in PCa. Finally, our findings suggest that therapeutic inhibition of CAV1, TGFβ signalling, or its upstream regulatory network, could be beneficial for PCa patients with CAV1 over-expression.

## Methods

### Patient samples and conventional IHC

Ethical approval for the use of clinical data and samples was obtained from the Institutional Ethics Committee of Hospital District of Helsinki and Uusimaa (D:no 446/13/03/02/2009), and by the Finnish agency for Health and Welfare (D:no THL490.5.05.00/2016) according to the national legislation. The use of the archived tissue blocks was approved by the National Supervisory Authority for Welfare and Health (VALVIRA, D:no 4076/32/300/02), giving us a permission to apply old diagnostic leftover samples in research in an unlinked fashion with no patient identifiers preserved. Given the time frame from diagnostic sample processing to application in research, patient consent for the use of such samples is not possible nor needed due to the VALVIRA permission. The experiments conformed to the principles set out in the WMA Declaration of Helsinki and the Department of Health and Human Services Belmont Report. All FFPE samples were obtained from the Department of Pathology at the Helsinki University Hospital (HUH). Formalin fixation and paraffin embedding were performed in the central laboratory of HUH according to standard procedures. The samples were anonymous and all unique identifiers, including photos, were removed from the published images and figure legends.

Study population, conventional chromogenic immunohistochemistry methods, and tissue microarray construction were previously published in detail^49^. The TMA_1 consisted of 435 patients (Table 1) with two 1 mm cores from the primary Gleason grade area (index lesion) and one from the secondary Gleason grade area. One core of each patient contained an adjacent benign area. The PCa TMA_2 consisted of 70 PCa patients with one 1 mm core from the cancer area and another one from the adjacent benign area.

### MicroArray datasets

#### Description of microarray datasets

The microarray data used in this study was acquired from three publicly available data repositories. We used each dataset at different stages of the study as described below:

1. GeneSapiens database^36^ was used for the analysis of mesenchymal and epithelial expression clusters. The microarray data in Genesapiens database was processed as described in the original article^36^. We selected 35 epithelial cell lines and 4 mesenchymal cell lines based on prior biological knowledge about these cell lines.
2. GSK300 microarray dataset was used for the analysis of CAV1 correlating genes. The original raw data was generated under GlaxoSmithKline Cancer cell line Genomic profiling project. This raw data was downloaded from Cancer Biomedical Informatics Grid (caBig) portal and all samples were profiled using Affymetrix Human Genome U133 Plus 2.0 Array Platform. The raw CEL files data was normalized using the Aroma Affymetrix (Version 1.3.0) R package^62^ (http://www.aroma-project.org) based on custom CDF files (version 16) found at http://brainarray.mbni.med.umich.edu.
3. Connectivity map (cmap) microarray dataset generated by Broad Institute (http://www.broadinstitute.org/cmap/) shows the transcriptional responses of genes upon drug treatment. We did not use the already processed data in cmap database because the rank-based procedure described in the original Connectivity Map paper shows systematic amplification of measurements for low expressed genes. Even small differences in low intensities, which contain mostly noise, are ranked, and this has a significant impact on the identification of differentially expressed genes. Hence, we downloaded the raw data files in original CEL-format and re-processed them using Aroma Affymetrix R package as described above^62^. We used expression profiles from the most abundant microarray platform (HT-HG-U133A) in this dataset.

### Microarray data analysis

Datasets 2 and 3 described above were RMA-normalized before computing differential expression using the aroma.affymetrix^62^. The intensity values from the probesets mapping to a single gene were combined to get a single intensity value per gene using Tukey biweight. The already normalized data from Genesapiens database were further processed using canonical correspondence analysis (cca) by performing multi-dimension reduction using ANOVA-PCA approach. Differentially expressed genes between the mesenchymal and epithelial group of cell lines were determined using the limma R/Bioconductor package. For the cmap dataset, we used the default Benjamini-Hochberg multiple testing correction method to filter out differentially expressed genes between un-treated and treated cell line data based on a q-value threshold (q < 0.05). We sorted the resulting data by the logarithmic fold change value (logFC) and took only genes with an absolute logFC > 1.5.

### Antibodies in IHC

CAV1 BD6100407 (clone 2297) (1/1000 for IHC, 1/300 fluorescence). ITGB1 (EP1041Y; Ab52971; 1/500 IHC, 1/100 fluorescence). E-cadherin from CST (24E10, 1/300 fluorescence). The integrin antibodies ITGA2 (Novus, NBP1-96715, 1/200) and ITGA6 (Novus, NBP1-85747, 1/200). The Pan-CK antibody (C-11, Abcam, ab7753, 1 to 1500) and the KRT5 antibody (Abcam, ab52635, 1 to 2000).

### Antibodies in Western blotting

Antibodies against Cav1 (D46G3; 1/2000), E-cadherin (24E10; 1/2000), Slug (C19G7; 1/1000), pSmad3 (S423/425, 9520; 1/250), and CDK6 (3136; 1/500) were from CST. Pai-1(sc-5297; 1/500), Smad2/3, (sc-8332; 1/500), p21 (sc-397; 1/500), and ITGB4 (sc-9090, 1/500) were from Santa-Cruz. Beta-tubulin (ab6046; 1/10 000), ITGB1 (EP1041Y; 1/5000), and PSF (ab38148; 1/1000) were from Abcam. Actin was from Sigma-Aldrich (A1978; 1/10 000), GAPDH from Millipore (MAB374; 1/3000), and DSP from Bethyl (A303-355A; 1/1000).

### Multiplexed IHC

After heat-induced epitope retrieval (HIER) (see details from ref.^35^), slides were incubated with primary antibody followed by washing and HRP-conjugated anti-mouse or anti-rabbit secondary antibodies (Immunologic, Netherlands). The HRP reaction was linked to Tyramide signal amplification (TSA) using tyramide-alexa-488 according to the manufacturer’s protocol (Life Technologies, TSA™ Reagent, Alexa Fluor® 488 Tyramide, T20948). Then, slides were heated as in HIER and incubated with two other primary antibodies. Visualization for other than TSA-linked detection was based on AlexaFluor-conjugated anti-mouse and anti-rabbit antibodies (AlexaFluor-555, AlexaFluor-647, 1 to 500, Life Technologies) and Hoechst 33342 (1 µg/ml) for nuclear signal. Slides were mounted using ProLong Gold antifade reagent (In vitrogen).

### Tissue analysis

For the conventional IHC, the visual inspection of CAV1 and ITGB1 expression (scale 0–3) in TMA_1 was performed blinded to clinicopathological parameters by T.P. and S.B. Inconsistencies were agreed together with a certified pathologist specialized in urological cancers (T.M.) to reach consensus. CAV1 and ITGB1 expression scores in multiple cancer cores were averaged and categorized for the final score of a patient. Only patients with two or more cancer cores were included in the final analysis (n = 435). Due to detachment of benign cores or false representation (stroma only or cancer present), the final patient number for benign samples was n = 401. Only patients who had prostatectomy as the primary treatment and known Gleason score or pathological tumour stage (pT) status, were included in the final analysis.

For fluorescent detection and quantification of CAV1 and E-cadherin, a MatLab-based image analytical algorithm was developed. For each patient, 3–4 representative image regions positive for pan-cytokeratin (panCK) were selected for quantification, and benign and cancer epithelial areas were detected based on KRT5 expression, which is usually lost in prostate adenocarcinoma. For final results, both CAV1 and E-cadherin channel intensities were measured as the average protein expression intensity α (α = (total channel intensity)/ (total area)). For CAV1 and integrin co-expression analysis in FFPE sections, ImageJ was applied as following: TMA spots were manually segmented from the background. The epithelium was segmented using panCK positivity and threshold was set using visual assessment. The stroma was defined as the inversion of the epithelial segmentation mask. Object areas were computed by default ImageJ watershed algorithm and particle analysis. Mean and median intensities were calculated within each object area, and for final results, areas larger than 100 pixels were included as true objects.

### RNAi

The cell lines, PNT2, Du145, and PC-3 from ATCC, were maintained in RPMI 1640 (Gibco, 11875-085) supplemented with 10% FBS (Gibco). siRNA-mediated cell silencing was done using Lipofectamine 2000 or RNAiMAX reagents (Life Technologies) using 1 to 1000 of the transfection reagent and 10 nM siRNA (final conc.). siRNAs were ON-TARGETplus siRNAs from Dharmacon. siRNAs used for experiments other than RNAi screen were the following: siCAV1:GAGCUUCCUGAUUGAGAUUdTdT; siCAV1_2: AAGAGCTTCCTGATTGAGATT; siCTRL: ON-TARGETplus Non-targeting siRNA #1 and #2; siITGB1: J-004506-05, J-004506-06, J-004506-07, J-004506-08; ITGA2: J-004566-06, J-004566-08; siITGA5: J-008003-08; siITGA6: J-007214-06; siKIND-2: J-012753-05; siFOSL1: J-004341-06, J-004341-07, J-004341-08), where the code is the dublex catalog number for Dharmacon siRNAs. Alternatively, CAV1 was silenced using a lentiviral system (L-shCAV1), where target sequence corresponded to human CAV1 nucleotides 254–277 (‘5-GACGTGGTCAAGATTGACTTT-3’). This sequence was cloned into pLVX-shRNA2, which contains a ZsGreen1 reporter (Clontech, Mountain View, CA). The use of this construct has been well-documented^63,64^. Lentiviral infection was performed as in^63^. The siRNA sequences used in the siRNA screen can be found in the Supplementary Table S8. The RNAi screen methodology was done as described in^65^.

### 3D invasion assays

The 3D invasion assays were done as described earlier^63,66^ with minor modifications. Briefly, 7000 cells were applied onto Ibidi (Angiogenesis) chambers, and after 3 h attachment, either Matrigel (50%) or Collagen type I (1.5 mg/ml, BD) was applied (25 µl) on top of the cells for 3 h, after which 25 µl of medium was added on top with daily addition of serum or TGFβ to create gradient. After incubation for 3 days (p21 determination) or 4 days (invasion assay), cells were either lysed for Western blotting or fixed for immunocytochemistry or IF. For lysis, collagen and Matrigel were first solubilized using 0.01 M HCL or cold PBS + 0.02 M EDTA, respectively, and cells were collected and lysed with Laemmli buffer. For IF, cells were fixed with 4% paraformaldehyde PBS (PFAH-PBS), permeabilised with Triton-X100 (0.3%) and stained using Hoechst 33342 and Phalloidin-647 (InVitrogen). Imaging was done using SP5 Leica confocal microscope with 20x objective by imaging the centre area of each well. Images were analysed using ImageJ. Invasiveness was quantified as the area invaded. Total number of cells at the end point was quantified by counting the nuclei in all of the acquired planes using 3D cell count plugin (ImageJ). For IHC, cells were fixed with 8% PFAH-PBS for 20 min, fixative inactivated with TRIS-EDTA (50 mM, 2 mM), and gel pieces collected for paraffin processing using Shandon Cytoblocks (Thermo Scientific, 7401150).

### MicroArray for CAV1 vs. Control silenced cells

To study the differential expression of genes in PC-3 cells silenced with shCAV1 or shCTRL, the following was done. RNA was purified from cells with stable knock-down of CAV1 (L-shCAV1) or from control cells (L-shCTRL) (3 days adherent cultures with passage 4 after transfection, 80% confluent when collecting, the experiment was done in three replicates). One-Color Microarray-Based Gene Expression Analysis Protocol (Agilent Technologies, Palo Alto, CA, USA) was used to amplify and label RNA. Briefly, 1000 ng of total RNA from different samples were reverse transcribed using T7 promoter primer and the Moloney murine leukemia virus (MMLV) reverse transcriptase (RT). cDNA was then converted to anti-sense RNA (aRNA) by using T7 RNA polymerase that amplifies target material and incorporates cyanine 3 (Cy3)-labeled CTP simultaneously. Samples were hybridized to a Whole Human Genome Microarray 4 × 44 K (G4112F, Agilent Technologies). 1.65 micrograms of Cy3-labeled aRNA was hybridized for 17 hours at 65 °C in Agilent hybridization oven (G2545A, Agilent Technologies) set to 10 rpm in a final concentration of 1 × GEx Hybridization Buffer HI-RPM (Agilent Technologies). Arrays were washed and dried out using a centrifuge according to manufacturer’s instructions (One-Color Microarray-Based Gene Expression Analysis, Agilent Technologies). Arrays were scanned at 5 mm resolution on an Agilent DNA Microarray Scanner (G2565BA, Agilent Technologies) using the default settings for 4 × 44 k format one-color arrays. Images provided by the scanner were analysed using Feature Extraction software v10.1.1.1 (Agilent Technologies).

For data analysis, raw signals were thresholded to 1 and quantiles normalization^67^ was performed using the software GeneSpring. Data were considered in the log2 scale. Default flags were considered as absent, except saturated points that were flagged as marginal. From the initial 41081 probes present in the Agilent 4 × 44 K chip, 23702 remained after applying three types of filter:

1. By expression
  Retain those genes where at least 100 percent of samples in any 1 out of 4 conditions have values within the accepted intensity range ([20,100] percentiles): 33064 probes remain.
2. By flags
  Retain those genes where at least 100 percent of samples in any 1 out of 4 conditions have reliable values (Present and Marginal Flags). 30838 probes remain.
3. By error

Retain only those genes that change across the experiment, i.e. with coefficient of variation (CV) > 1% across all samples. 23702 probes remain.

### Quantitative reverse transcriptase PCR

Total RNA was extracted using the RNeasy plus mini kit (Qiagen 74134) and the cDNA was obtained from 1 ug of total RNA by using an Omniscript RT kit (Qiagen 205111). Quantitative PCR was carried out in a 7900 HT Fast Real-time PCR System (Applied Biosytems) using a power SYBR Green PCR master mix (Applied biosytems 4367659) and the following specific primer sets with FOR and REV primers, respectively: 5′-atccacagctgtcatagtc-3′ and 5′-cacttggcccatgaaaag-3′ for SERPINE1; 5′-cagtgattatttccccgtatc-3′ and 5′-ccccaaagatgaggagtatc-3′ for SNAI2 (SLUG); 5′-gagagagaggaaggagattc-3′ and 5′-gagtttctccctgaaatgtg-3′ for EDN1; 5′-aggcacagatgttaaagatg-3′ and 5′-ccgagatattatttctgcatgg-3′ for ADARB1; 5′-aactaacttctcccccatac-3′ and 5′-cttaaaaggctctgccttag-3′ for OAS2; 5′-ttgtatactaccatagtgagcc-3′ and 5′-tttggagaaaacagaacacc-3 for AGR2; 5- gacaagaagtactaccccag-3 and 5-gagatcaagggaatgttcaag for BST2. For normalization of the signals, ACTIN 5′-caccttccagcagatgtcga-3′ and 5′-agcatttgcggtggatgg-3′, as well as GAPDH 5′-atcaccatcttccaggagcg-3′ and 5′-cctgcaaatgagccccag-3′ were used. The PCR program applied 40x cycles of 95°10 min/95°15 s, 60°1 min/95°15 s, 60°15 s, and 95. Biogazelle qbaseplus program was used to analyze and normalize the data.

### Statistics

The normality of data was tested using Shapiro-Wilk test. For normally distributed data, Student´s t-test (two-sided) was employed to compare mean expression between groups. For non-normally distributed data, Welch’s one-way ANOVA was employed to compare mean expression between groups. We used Pearson Chi-square to test significance of cross-tabulations for nominal and categorical data. Spearman rho was used for correlation analysis between categorical variables. For microarray gene expression analysis extracted from databases, we performed correlation analysis of CAV1 across the whole transcriptome of genes using the Pearson correlation method across over 300 cell line samples in the GSK300 dataset. We selected the top 500 genes having a correlation score (r > 0.3) for gene set enrichment analysis in the David gene ontology analysis portal. The limma package from Bioconductor was used for cDNA microarray statistics^68^. Limma calculates moderated t-statistics, adding to the error term some information on the variance of all genes, solving the typical microarray problem of small sample size. For tissue image analysis and for RNAi cell screen image analysis standard two-sided (two-tailed) Student’s *t* test was used. P-values <0.05 were considered statistically significant.

## Electronic supplementary material

Supplementary information
