# Supplementary material for: ITGB1-dependent upregulation of Caveolin-1 switches TGFβ signalling from tumour-suppressive to oncogenic in prostate cancer
Source: Sci Rep. 2018 Feb 5;8:2338. doi: 10.1038/s41598-018-20161-2 (PMC5799174; doi:10.1038/s41598-018-20161-2)

SUPPLEMENTARY INFORMATION

ITGB1-dependent upregulation of Caveolin-1 switches TGFβ signalling from tumour-suppressive to oncogenic in prostate cancer

Teijo Pellinen, Sami Blom, Sara Sánchez, Katja Välimäki, John-Patrick Mpindi, Hind Azegrouz, Raffaele Strippoli, Raquel Nieto, Mariano Vitón, Irene Palacios, Riku Turkki, Yinhai Wang, Miguel Sánchez-Alvarez, Stig Nordling , Anna Bützow, Tuomas Mirtti, Antti Rannikko, María C. Montoya, Olli Kallioniemi, and Miguel A. del Pozo

Supplementary Tables (Table S1-S8)

Table S1: Epithelial and mesenchymal cell lines

Table S2: Top 100 mesenchymal genes listed in the logFC rank order

Table S3: Top 100 epithelial genes in the logFC rank order

Table S4: CAV1 silencing effects on known TGFβ target genes

Table S5: CAV1 silencing effects on top mesenchymal or epithelial genes

Table S6: GoTerm categories of top 500 genes with highest correlation to CAV1 mRNA expression in 300 cell lines

Table S7: Genes selected for RNAi screen

Table S8: siRNAs used in the RNAi screen

Supplementary Figures (Figure S1-S5)

Figure S1: CAV1 and E-cadherin show inverse association both at the inter-patient and intra-patient level in PCa

Figure S2: Gene network emerging from genes significantly impacted by CAV1 knockdown

Figure S3: CAV1 regulates TGFβ target genes

Figure S4: CAV1 expression regulation by beta1 integrins

Figure S5: CAV1 is co-expressed with beta1 integrins in clinical prostate cancer

Supplementary References

Original full scans of Western blots

Supplementary Tables S1-S8

Table S1. **Epithelial and mesenchymal cell lines**. The microarray data was extracted from GeneSapiens database (version 1) [1].

Epithelial cell line	Tissue type	Mesenchymal cell line	Tissue type
1013L	prostate	D551 (Detroit)	skin fibroblast
1A9	ovary	WI-38	lung fibroblast
22RV1	prostate	WJ1	umbilical cord mesench
A549	lung	WS1	skin fibroblast
BT-474	breast		
DU145	prostate		
DuCaP	prostate		
EP156T	prostate		
HCC1569	breast		
HCC1954	breast		
HCC202	breast		
JIMT-1	breast		
KF28	ovary		
KFr13	ovary		
KPL4	breast		
LNCaP	prostate		
LNCaP_USA	prostate		
MCF10A	breast		
MCF7	breast		
MCF7_ICLC	breast		
MDA-MB-231_atcc	breast		
MDA-MB-231_clean	breast		
MDA-MB-453	breast		
MDA-MB-468	breast		
MDa-Pca2a	prostate		
NCI-H660	prostate		
PC-3	prostate		
PSK1	prostate		
SKBR3	breast		
SUM190	breast		
SUM206	breast		
SUM225	breast		
T47D	breast		
VcaP	prostate		
ZR751	breast		

Table S2. **Top 100 mesenchymal genes listed in the logFC rank order**.

GENE ID	logFC	p-value
COL3A1	-7.88	0.00
GREM1	-7.61	0.00
COL6A3	-7.16	0.00
SPARC	-7.12	0.00
COL1A2	-6.55	0.00
KIAA1913	-6.42	0.00
PTX3	-6.21	0.00
DKK3	-6.15	0.00

PDGFRA	-5.99	0.00
TAGLN	-5.95	0.00
POSTN	-5.93	0.00
CTGF	-5.71	0.00
FBN1	-5.66	0.00
MMP2	-5.66	0.00
GJA1	-5.65	0.00
MMP1	-5.64	0.00
LOX	-5.63	0.00
NID2	-5.53	0.00
COL5A2	-5.49	0.00
SULF1	-5.41	0.00
FAP	-5.30	0.00
SERPINE1	-5.29	0.00
SERPINE2	-5.27	0.00
LRRC17	-5.23	0.00
TGFBI	-5.22	0.00
COL5A1	-5.22	0.00
EMP3	-5.09	0.00
GLT8D2	-5.09	0.00
COL4A1	-5.04	0.00
TGFB1I1	-5.03	0.00
PLAT	-4.98	0.00
SRGN	-4.95	0.00
NNMT	-4.94	0.00
VCAN	-4.84	0.00
MYL9	-4.83	0.00
SPOCK1	-4.81	0.00
TNFAIP6	-4.75	0.00
C2orf32	-4.74	0.00
NEXN	-4.72	0.00
THBS2	-4.70	0.00
TCEAL7	-4.68	0.00
VIM	-4.68	0.00
GNG11	-4.65	0.00
SRPX	-4.64	0.00
WNT5A	-4.61	0.00
SNAI2	-4.60	0.00
CTHRC1	-4.59	0.00
LTBP2	-4.59	0.00
PRKCDBP	-4.57	0.00
LOXL1	-4.54	0.00
TNC	-4.47	0.00
COL4A2	-4.42	0.00
TMEM47	-4.41	0.00

FEZ1	-4.33	0.00
CDH11	-4.32	0.00
ACTA2	-4.29	0.00
HTRA1	-4.24	0.00
AKR1B1	-4.22	0.01
DCN	-4.22	0.00
FBLN5	-4.21	0.00
HMCN1	-4.19	0.00
LOXL2	-4.16	0.00
ITGBL1	-4.12	0.00
TFPI2	-4.12	0.00
RECK	-4.10	0.00
EFEMP1	-4.10	0.00
CAV1	-4.09	0.01
PTGS2	-4.09	0.00
ENSG00000166250	-4.09	0.00
COL6A2	-4.05	0.00
COL1A1	-4.05	0.00
THY1	-4.03	0.00
COL12A1	-4.02	0.00
FN1	-4.02	0.00
MFAP2	-4.00	0.00
COL8A1	-3.95	0.00
TRIM22	-3.94	0.00
RFTN1	-3.92	0.00
ENSG00000169184	-3.92	0.00
SCG5	-3.91	0.00
ENSG00000137463	-3.91	0.00
RGS4	-3.88	0.00
CCDC80	-3.86	0.00
IGFBP6	-3.85	0.00
TRHDE	-3.85	0.00
LHFP	-3.84	0.00
ENSG00000235531	-3.79	0.00
GEM	-3.78	0.00
GLIPR1	-3.78	0.00
NID1	-3.77	0.00
PCDH18	-3.77	0.00
DSE	-3.76	0.00
FGF2	-3.76	0.00
PRR16	-3.76	0.00
EFEMP2	-3.74	0.00
PCOLCE	-3.74	0.00
ENSG00000149090	-3.72	0.00
ALPK2	-3.72	0.00

IFI16	-3.72	0.00
ENSG00000182667	-3.72	0.00

Table S3. **Top 100 epithelial genes in the logFC rank order**.

GENE ID	logFC	p-value
EPCAM	7.01	0
MAL2	6.68	0
TPD52	5.86	0
RBM35A	5.35	2e-05
SPINT2	4.83	0
S100P	4.58	0.00
GPR160	4.32	5e-05
CDH1	4.13	4e-05
VAMP8	4.06	9e-05
ELOVL7	3.95	1e-05
RAB25	3.84	0.00
MYO5C	3.80	0
CHMP4C	3.78	0
KRT19	3.61	0.02
TACSTD2	3.55	0.00
CTSH	3.49	1e-05
AZGP1	3.40	0.02
LSR	3.36	0
GRHL2	3.34	0.00
TOX3	3.15	0.02
PRRG4	3.15	9e-05
AGR2	3.14	0.01
PLS1	3.12	0
CLDN7	3.10	3e-05
HOOK1	3.10	2e-05
MTAC2D1	3.09	0.00
CDS1	3.07	5e-05
C10orf57	3.06	0.00
FOXA1	2.99	0.00
MAP7	2.99	1e-05
CXADR	2.99	2e-05
INHBB	2.98	0.01
S100A14	2.97	0.02
ENPP5	2.94	0.00
GALNT3	2.92	0.00
SNX10	2.92	3e-05
EEF1A2	2.91	0.01
CLDN3	2.89	0.00

ELF3	2.87	2e-05
TMEM45B	2.86	0.00
SFN	2.85	0.00
EFNA1	2.85	6e-05
NMU	2.76	0.00
ST14	2.76	5e-05
EXPH5	2.74	2e-05
BLNK	2.74	0.00
FAM110C	2.74	0.00
MYO5B	2.74	0
FGF13	2.71	0.01
ZNF165	2.71	5e-05
FXYD3	2.70	0.00
GCA	2.70	0.00
KIAA1244	2.68	0.00
RHPN2	2.68	0
MARVELD2	2.65	1e-05
IRX3	2.64	0.01
PRSS8	2.62	0.00
RBM35B	2.61	3e-05
IGFBP2	2.60	0.01
KIF21A	2.60	0
AP1M2	2.60	1e-05
ZIC2	2.60	0.00
SLCO4A1	2.60	0.00
DSC2	2.57	2e-05
F11R	2.57	1e-05
GRHL1	2.56	0.00
GATA3	2.55	0.03
C9orf152	2.54	0.02
TSPAN13	2.53	0.00
SPDEF	2.46	0.00
RASEF	2.46	0.00
USP43	2.46	1e-05
FAM84B	2.45	0
SLC27A2	2.45	0.00
TFAP2C	2.43	0.00
ENSG00000163694	2.41	0
EPN3	2.40	0.01
CLGN	2.39	0.03
GCH1	2.38	1e-05
SLC16A14	2.38	0.04
KIAA1598	2.38	0.00
ZDHHC23	2.37	0
CREB3L4	2.36	0.00

ENSG00000160588	2.34	7e-05
C3orf57	2.34	0.04
BST2	2.33	0.01
LCN2	2.32	0.05
ATAD4	2.32	0.00
ATAD2	2.31	6e-05
TMC4	2.31	0.00
TM7SF2	2.29	0.00
PPL	2.29	0.01
SYCP2	2.29	0.03
ENSG00000204832	2.29	0.02
OVOL2	2.28	0.00
LLGL2	2.27	5e-05
TNFSF10	2.27	0.04
SYTL1	2.26	0.00
ZNF367	2.25	0.00
SPINT1	2.25	0

Table S4. CAV1 silencing effects on known TGFβ target genes.

**DOWN-REGULATION by CAV1 silencing**					
**Gene**	**Cluster[a]**	**logFC[b]**	**p-value**	**TGFβ effect**	**Reference for TGFβ effect**
SERPINE1	mesenchymal	-1.77	1.30E-04	UP	[2,3,4]
TP53I11		-1.76	0.018	DOWN	[5]
SLC7A4		-1.54	0.008	UP	[2]
ADARB1		-1.52	1.25E-04	DOWN	[6]
BDNF	mesenchymal	-1.51	0.003	UP	[6]
RNF152		-1.5	0.029	UP	[6]
NDST1		-1.4	1.48E-04	UP	[6]
CDK6		-1.37	1.05E-04	UP	[5]
CDH6	mesenchymal	-1.34	0.00201	UP	[7]
VCAN	mesenchymal	-1.33	1.02E-04	UP	[8,9,10]
RUNX1T1		-1.33	2.14E-04	UP	[6]
HMGN2		-1.32	7.18E-05	DOWN	[10,11]
KITLG		-1.24	0.004	UP	[6]
CCND1		-1.12	7.66E-04	UP	[2]
NRP2	mesenchymal	-1.1	0.001	UP	[5]
G0S2		-1.1	1.22E-04	UP	[2]
SNAI2	mesenchymal	-0.87	0.004	UP	[2,3]
PDGFRA	mesenchymal	-0.87	2.34E-04	DOWN	[2,12]
EDN1		-0.84	8.12E-04	DOWN/ UP	[2]
TGM2	mesenchymal	-0.82	0.003	UP	[5]

FHL2		-0.74	0.44E-04	UP	[3]
CTGF	mesenchymal	-0.75	0.002	UP	[4,12]
SRF		-0.72	1.75E-04	UP	[13,14]
TGFB1I1	mesenchymal	-0.34	0.028	UP	[14]
**UP-REGULATION by CAV1 silencing**					
**Gene**	**Cluster**	**logFC**	**p-value**	**TGFβ effect**	**Reference for TGFβ effect**
IRF7		1.27	2.36E-04	DOWN	[2]
IL1B	mesenchymal	1.29	3.04E-04	DOWN	[2,4]
IFI35		1.34	1.58E-04	DOWN	[11]; GSE17708
LOXL4	mesenchymal	1.47	6.81E-05	UP	[15]
AGR2	epithelial	1.48	8.12E-05	DOWN	[16]
SERPINB2		1.54	2.34E-04	DOWN	[2]
FGL2	mesenchymal	1.71	0.012	DOWN	[2]
S100P	epithelial	1.74	7.64E-05	UP/DOWN	[2,16]
ELF3	epithelial	1.84	2.15E-04	DOWN	[17]
DSP		1.93	2.61E-05	DOWN	[2,18]
ITGB4		1.97	6.05E-05	DOWN	[2]
IFIT1		1.99	2.61E-05	DOWN	[2]
CDH1	epithelial	2.15	2.34E-04	DOWN	[18]
PRR15		2.2	3.77E-04	DOWN	[11]; GSE17708
HPGD		2.52	2.65E-05	UP	[19]
HERC6	epithelial	2.59	9.52E-05	DOWN	[6]
OAS2		2.83	2.61E-05	DOWN	[20]
MX1		3.05	4.42E-06	DOWN	[5]
EPSTI1	mesenchymal	3.72	3.57E-06	DOWN	[6]
OAS1	epithelial	3.82	3.57E-06	DOWN	[6]
IFITM1		4.48	3.57E-06	DOWN	[5]
BST2	epithelial	5.944445	3.57E-06	DOWN	[21]

[a]gene marked as mesenchymal or epithelial when significantly clustering to these groups according to the analysis in Figure 1.
[b]logFC = logarithmic fold change of expression (CAV1 KD vs. CTRL KD).

Table S5. **CAV1 silencing effects on top mesenchymal or epithelial genes**. Changes in the expression of top 25 mesenchymal and epithelial genes upon CAV1 silencing.

**Top mesenchymal**	**logFC[a]**	**p-value**	**Significant change (UP/DOWN)**	**Top epithelial**	**logFC**	**p-value**	**Significant change (UP/DOWN)**
COL3A1	-0.5	0	DOWN	EPCAM	0.23	0.07	
GREM1	-0.02	0.94		MAL2	1.53	0	UP
COL6A3	-0.2	0.04	DOWN	TPD52	-0.21	0.08	
SPARC	0.58	0	DOWN	RBM35A	0.3	0.02	UP

COL1A2	-0.05	0.68		SPINT2	0.78	0	UP
KIAA1913	-0.57	0	DOWN	S100P	1.74	0	UP
PTX3	0.11	0.22		GPR160	0	0.99	
DKK3	-0.29	0.03	DOWN	CDH1	2.15	0	UP
PDGFRA	-0.87	0	DOWN	VAMP8	0.74	0	UP
TAGLN	na	na		ELOVL7	na	na	
POSTN	na	na		RAB25	na	na	
CTGF	-0.41	0	DOWN	MYO5C	0.33	0.01	UP
FBN1	0.27	0.01	UP	CHMP4C	0.41	0.01	UP
MMP2	na	na		KRT19	na	na	
GJA1	-0.27	0.03	DOWN	TACSTD2	0.08	0.33	
MMP1	-0.42	0.01	DOWN	CTSH	0.28	0.01	UP
LOX	-0.13	0.15		AZGP1	0.14	0.73	
NID2	-0.27	0.02	DOWN	LSR	0.76	0	UP
COL5A2	0.49	0	UP	GRHL2	na	na	
SULF1	0.17	0.25		TOX3	0.06	0.93	
FAP	na	na		PRRG4	0.24	0.01	UP
SERPINE1	-1.77	0	DOWN	AGR2	1.48	0	UP
SERPINE2	0.24	0.02	UP	PLS1	0.28	0.02	UP
LRRC17	na	na		CLDN7	0.4	0.01	UP
TGFBI	na	na		HOOK1	0.27	0.05	UP

[a]logFC = logarithmic fold change of expression (CAV1 KD vs. CTRL KD).

Table S6. **GoTerm categories of top 500 genes with highest correlation to CAV1 mRNA expression in 300 cell lines**.

**Biological process**	count	percent	p-value
cell adhesion	64	12.9	6.9E-16
biological adhesion	64	12.9	7.3E-16
regulation of cell motion	31	6.3	4.8E-14
regulation of cell migration	27	5.5	3.2E-12
cell-substrate adhesion	20	4.0	4.5E-11
regulation of locomotion	27	5.5	6.1E-11
cell motion	43	8.7	1.2E-10
positive regulation of cell motion	18	3.6	2.9E-9
cell-matrix adhesion	17	3.4	5.0E-9
enzyme linked receptor protein signalling pathway	33	6.7	5.7E-9

**Cellular component**	count	percent	p-value
cell-substrate junction	27	5.5	4.1E-16
adherens junction	31	6.3	5.6E-16
basolateral plasma membrane	34	6.9	3.7E-15

focal adhesion	25	5.1	4.6E-15
anchoring junction	31	6.3	9.8E-15
cell-substrate adherens junction	25	5.1	1.2E-14
cell leading edge	25	5.1	5.3E-12
basement membrane	18	3.6	1.8E-10
plasma membrane	176	35.6	2.6E-10
extracellular matrix part	21	4.2	4.4E-10

**Molecular function**	count	percent	p-value
cytoskeletal protein binding	50	10.1	4.2E-14
actin binding	38	7.7	6.8E-13
protein complex binding	24	4.8	9.6E-9
integrin binding	13	2.6	8.5E-8
extracellular matrix binding	9	1.8	5.8E-7
protein dimerization activity	37	7.5	2.3E-6
identical protein binding	41	8.3	2.8E-6
calcium ion binding	51	10.3	8.0E-6
protein homodimerization activity	26	5.3	1.2E-5
actin filament binding	10	2.0	1.7E-5

Table S7. Genes selected for RNAi screen.

Genes selected among the top 500 correlators with CAV1 expression in 300 cell lines

Gene symbol	Ensemble code	corr	corr rank	corr pval
CAV1	ENSG00000105974	1	1	0
MYOF	ENSG00000138119	0.67	3	0
ANXA2	ENSG00000182718	0.66	4	0
ITGA3	ENSG00000005884	0.64	6	0
CAPN2	ENSG00000162909	0.63	7	0
FRMD6	ENSG00000139926	0.61	8	0
FOSL1	ENSG00000175592	0.6	10	0
PTRF	ENSG00000177469	0.6	11	0
ANLN	ENSG00000011426	0.57	23	0
ACTN1	ENSG00000072110	0.56	25	0
PXN	ENSG00000089159	0.56	27	0
CD59	ENSG00000085063	0.56	29	0
RIN2	ENSG00000132669	0.56	30	0
CD44	ENSG00000026508	0.55	32	0
LMNA	ENSG00000160789	0.53	48	0
RRAS	ENSG00000126458	0.53	51	0
EPHA2	ENSG00000142627	0.52	60	0
FLNA	ENSG00000196924	0.51	70	0
MET	ENSG00000105976	0.48	98	0

TGFB1I1	ENSG00000140682	0.48	102	0
LAMC1	ENSG00000135862	0.47	111	0
DKK1	ENSG00000107984	0.47	112	0
PLAUR	ENSG00000011422	0.47	118	0
PARVA	ENSG00000197702	0.46	126	0
FN1	ENSG00000115414	0.46	137	0
ITGA5	ENSG00000161638	0.46	138	0
PALLD	ENSG00000129116	0.45	163	0
LAMB1	ENSG00000091136	0.43	195	0
HIF1A	ENSG00000100644	0.42	209	0
RAB34	ENSG00000109113	0.42	221	0
ITGAV	ENSG00000138448	0.41	230	0
ITGA2	ENSG00000164171	0.41	238	0
JUN	ENSG00000177606	0.39	285	0
FLNC	ENSG00000128591	0.37	341	0
ITGA1	ENSG00000152684	0.37	381	0
ITGA6	ENSG00000091409	0.35	447	0

Adhesion genes selected outside the top 500 correlating genes

Gene symbol	Ensemble code	corr	corr rank	corr pval
NR3C1	ENSG00000113580	0.34	508	0
FERMT2	ENSG00000073712	0.28	870	0
LAMA4	ENSG00000112769	0.28	884	0
COL13A1	ENSG00000197467	0.25	1103	5.77E-15
LAMB2	ENSG00000172037	0.24	1211	1.11E-13
TLN1	ENSG00000137076	0.2	1583	4.68E-10
FHL1	ENSG00000022267	0.17	1983	1.78E-07
LAMA2	ENSG00000196569	0.09	3116	0
TWIST1	ENSG00000122691	0.09	3186	0
ITGB3	ENSG00000056345	0.09	3277	0.01
YWHAG	ENSG00000170027	0.08	3504	0.02
ETS1	ENSG00000134954	0.01	5189	0.83
AR	ENSG00000169083	-0.01	5573	0.84
ITGA7	ENSG00000135424	-0.09	9282	0
SRF	ENSG00000112658	-0.11	10032	0
CAV3	ENSG00000182533	-0.13	11218	9.37E-05
CSK	ENSG00000103653	-0.3	16961	3.69E-21
ITGB1	ENSG00000150093	N/A	N/A	N/A

Table S8. siRNAs used in the RNAi screen.

Gene Symbol	Duplex Catalog Number	GENEID	Gene Accession	GINumber	Sequence
DKK1	J-003843-12	22943	NM_012242	61676924	UAGAAAUAUUCCAGCGUUG
DKK1	J-003843-11	22943	NM_012242	61676924	AGGUCUGUCUUGCCGGAUA
DKK1	J-003843-10	22943	NM_012242	61676924	ACUGAUGAGUACUGCGCUA
DKK1	J-003843-09	22943	NM_012242	61676924	GUUCUCAAUUCCAACGCUA
COL13A1	J-012906-12	1305	NM_080815	22027608	UCAACUGCUGGACGAGAAA
COL13A1	J-012906-11	1305	NM_080815	22027608	CCAUUGGGCUGGACGGCAA
COL13A1	J-012906-10	1305	NM_080815	22027608	GAAAAGGGACCUCGCGGUA
COL13A1	J-012906-09	1305	NM_080815	22027608	GGGAGAAGCAGGUGUCGAU
FRMD6	J-016437-12	122786	NM_152330	34303929	ACUUACAGGACGAUGAAAU
FRMD6	J-016437-11	122786	NM_152330	34303929	AAGAAGCAGUACCGGGAAU
FRMD6	J-016437-10	122786	NM_152330	34303929	GGUGGAGAGUGGCGGCAAA
FRMD6	J-016437-09	122786	NM_152330	34303929	CAGCAAGGGUAUCGACCAA
FHL1	J-015857-05	2273	NM_001449	34147646	GGAAUCACUUACCAGGAUC
FHL1	J-015857-06	2273	NM_001449	34147646	GGACAAUCCUGGCACGACU
FHL1	J-015857-07	2273	NM_001449	34147646	GAAGUGUGCUGGAUGCAAG
FHL1	J-015857-08	2273	NM_001449	34147646	UGUGUUACCUGCUCUAAGA
ANXA2	J-010741-07	302	NM_004039	50845389	CGACGAGGACUCUCUCAUU
ANXA2	J-010741-08	302	NM_004039	50845389	AUCCAAGUGUCGCUAUUUA
ANXA2	J-010741-09	302	NM_004039	50845389	AAAACCAGCUUGCGAAUAA
ANXA2	J-010741-10	302	NM_004039	50845389	GGAAGAAAGCUCUGGGACU
ITGB3	J-004124-08	3690	NM_000212	47078291	GCAGUGAAUUGUACCUAUA
ITGB3	J-004124-09	3690	NM_000212	47078291	GAAGAACGCGCCAGAGCAA
ITGB3	J-004124-10	3690	NM_000212	47078291	GCCAACAACCCACUGUAUA
ITGB3	J-004124-11	3690	NM_000212	47078291	CCAGAUGCCUGCACCUUUA
CSK	J-003110-13	1445	NM_004383	4758077	GAAAUUCUCCACUAAGUCU
CSK	J-003110-12	1445	NM_004383	4758077	UCAAGUGCAUUAAGAACGA
CSK	J-003110-11	1445	NM_004383	4758077	ACGAGGAGGUGUACUUUGA
CSK	J-003110-10	1445	NM_004383	4758077	GCGAGUGCCUUAUCCAAGA
PXN	J-005163-08	5829	NM_002859	4506344	UGACGAAAGAGAAGCCUAA
PXN	J-005163-07	5829	NM_002859	4506344	CCAAACGGCCUGUGUUCUU
PXN	J-005163-06	5829	NM_002859	4506344	GGACGUGGCACCCUGAACA
PXN	J-005163-05	5829	NM_002859	4506344	CAACUGGAAACCACACAUA
RIN2	J-008173-12	54453	NM_018993	35493905	ACGCAAGGUAGCUGAGGUU
RIN2	J-008173-11	54453	NM_018993	35493905	UAACAAACAUGGGAACGUA
RIN2	J-008173-10	54453	NM_018993	35493905	AAGAUGUAUUCGCCGGAAA
RIN2	J-008173-09	54453	NM_018993	35493905	GGUCUGGACAAGCGAGGAA
ITGAV	J-004565-07	3685	NM_002210	40217844	CCUCUGACAUUGAUUGUUA

ITGAV	J-004565-08	3685	NM_002210	40217844	CCGAAACAAUGAAGCCUUA
ITGAV	J-004565-09	3685	NM_002210	40217844	GAACAUGUCCUCCUUAUAC
ITGAV	J-004565-10	3685	NM_002210	40217844	GUUCACGCCUGCUAACAUU
ETS1	J-003887-05	2113	NM_005238	41393580	AUAGAGAGCUACGAUAGUU
ETS1	J-003887-06	2113	NM_005238	41393580	GAAAUGAUGUCUCAAGCAU
ETS1	J-003887-07	2113	NM_005238	41393580	GUGAAACCAUAUCAAGUUA
ETS1	J-003887-08	2113	NM_005238	41393580	CAGAAUGACUACUUUGCUA
ITGA2	J-004566-06	3673	NM_002203	6006008	GAACGGGACUUUCGCAUCA
ITGA2	J-004566-07	3673	NM_002203	6006008	GAAACGCCCUUGAUACUAA
ITGA2	J-004566-08	3673	NM_002203	6006008	GUUCAGACCUACUAAGCAA
ITGA2	J-004566-09	3673	NM_002203	6006008	AAACAAGGCUGAUAAUUUG
ITGA6	J-007214-05	3655	NM_000210	4557674	GGAUCGAGUUUGAUAACGA
ITGA6	J-007214-06	3655	NM_000210	4557674	GGAUAUGCCUCCAGGUUAA
ITGA6	J-007214-07	3655	NM_000210	4557674	GAAAGGGAUUGUUCGUGUA
ITGA6	J-007214-08	3655	NM_000210	4557674	ACAGAUAGAUGAUAACAGA
FLNC	J-011272-05	2318	NM_001458	66279525	GAACAAGCAUUCUCUGUGA
FLNC	J-011272-06	2318	NM_001458	66279525	UGACAAGGAUCGCACCUAU
FLNC	J-011272-07	2318	NM_001458	66279525	UCUCAAGGGUGGACUGGUA
FLNC	J-011272-08	2318	NM_001458	66279525	GCAAACAGACGCCCAAGCA
JUN	J-003268-13	3725	NM_002228	44890066	UGAAAGCUCAGAACUCGGA
JUN	J-003268-12	3725	NM_002228	44890066	GAAACGACCUUCUAUGACG
JUN	J-003268-11	3725	NM_002228	44890066	GAACAGGUGGCACAGCUUA
JUN	J-003268-10	3725	NM_002228	44890066	GAGCGGACCUUAUGGCUAC
MET	J-003156-16	4233	NM_000245	42741654	GUAAGUGCCCGAAGUGUAA
MET	J-003156-15	4233	NM_000245	42741654	GAGCCAGCCUGAAUGAUGA
MET	J-003156-14	4233	NM_000245	42741654	GAACAGCGAGCUAAAUAUA
MET	J-003156-13	4233	NM_000245	42741654	GAACUGGUGUCCCGGAUAU
RRAS	J-010352-11	6237	NM_006270	20127497	CGACUUCCCCGUUGUGUUG
RRAS	J-010352-10	6237	NM_006270	20127497	AGUCCUACUUCGUGUCUGA
RRAS	J-010352-09	6237	NM_006270	20127497	GAACAAGAGCUCCCACCGA
RRAS	J-010352-08	6237	NM_006270	20127497	ACGAAGAUCUGCAGUGUGG
PLAUR	J-006388-08	5329	NM_001005377	53829380	GGACAGGACCUCUGCAGGA
PLAUR	J-006388-07	5329	NM_001005377	53829380	CGAGGUUGUGUGUGGGUUA
PLAUR	J-006388-06	5329	NM_001005377	53829380	CAUCAGACAUGAGCUGUGA
PLAUR	J-006388-05	5329	NM_001005377	53829380	CGGACUGGCUUGAAGAUCA
CAPN2	J-005804-05	824	NM_001748	12408645	CUACCAAGCUGUCGAUGAU
CAPN2	J-005804-06	824	NM_001748	12408645	CCGAGGAGGUUGAAAGUAA
CAPN2	J-005804-07	824	NM_001748	12408645	GGAACUACCCGAACACAUU
CAPN2	J-005804-08	824	NM_001748	12408645	UGGAAACGCUAUUCAAGAU
FOSL1	J-004341-08	8061	NM_005438	34734076	GAGUAAGGCGCGAGCGGAA
FOSL1	J-004341-07	8061	NM_005438	34734076	AAUCUGGGCUGCAGCGAGA

FOSL1	J-004341-06	8061	NM_005438	34734076	GAGCUGCAGUGGAUGGUAC
FOSL1	J-004341-05	8061	NM_005438	34734076	GCUCAUCGCAAGAGUAGCA
LAMB1	J-011713-05	3912	NM_002291	4504950	GGUGAUAUUUCGUGCUUUA
LAMB1	J-011713-06	3912	NM_002291	4504950	GGAUUUCUACCAUGAUUUA
LAMB1	J-011713-07	3912	NM_002291	4504950	GUGCUUGGCUGGUUACUAU
LAMB1	J-011713-08	3912	NM_002291	4504950	UAACUUCGAUUGAGUCUGA
ACTN1	J-011195-05	87	NM_001102	12025669	GAGACAGCCGACACAGAUA
ACTN1	J-011195-06	87	NM_001102	12025669	UGACUUACGUGUCUAGCUU
ACTN1	J-011195-07	87	NM_001102	12025669	GAACUGCCCGACCGGAUGA
ACTN1	J-011195-08	87	NM_001102	12025669	GAAUACGGCUUUUGACGUG
LMNA	J-004978-08	4000	NM_005572	27436944	CGUGUGCGCUCGCUGGAAA
LMNA	J-004978-07	4000	NM_005572	27436944	UGAAAGCGCGCAAUACCAA
LMNA	J-004978-06	4000	NM_005572	27436944	UCACAGCACGCACGCACUA
LMNA	J-004978-05	4000	NM_005572	27436944	GAAGGAGGGUGACCUGAUA
NR3C1	J-003424-10	2908	NM_001020825	66528641	GCAUGUACGACCAAUGUAA
NR3C1	J-003424-09	2908	NM_001020825	66528641	UGACAAAACUCUUGGAUUC
NR3C1	J-003424-08	2908	NM_001020825	66528641	GGAAACAGACUUAAAGCUU
NR3C1	J-003424-07	2908	NM_001020825	66528641	GAACUUCCCUGGUCGAACA
FN1	J-009853-06	2335	NM_054034	47132546	GCAGCACAACUUCGAAUUA
FN1	J-009853-07	2335	NM_054034	47132546	GAAAUAGAUGCAACGAUCA
FN1	J-009853-08	2335	NM_054034	47132546	GAGGAAAUCUGCACAACCA
FN1	J-009853-09	2335	NM_054034	47132546	CCUAAAGACUCCAUGAUCU
TGFB1I1	J-006565-07	7041	NM_015927	34147679	GCAACCAGCCCAUCCGACA
TGFB1I1	J-006565-08	7041	NM_015927	34147679	GAAAUCAUGUCUCAGUUCC
TGFB1I1	J-006565-09	7041	NM_015927	34147679	GAGCGCUUCUCGCCAAGAU
TGFB1I1	J-006565-10	7041	NM_015927	34147679	AGGACCAGUCUGAAGAUAA
FLNA	J-012579-05	2316	NM_001456	4503744	GCAGGAGGCUGGCGAGUAU
FLNA	J-012579-06	2316	NM_001456	4503744	GCACCCAGACCGUCAAUUA
FLNA	J-012579-07	2316	NM_001456	4503744	GCACAUGUUCCGUGUCCUA
FLNA	J-012579-08	2316	NM_001456	4503744	GAAUGGCGUUUACCUGAUU
TLN1	J-012949-05	7094	NM_006289	16753232	GAAGAUGGUUGGCGGCAUU
TLN1	J-012949-06	7094	NM_006289	16753232	GUAGAGGACCUGACAACAA
TLN1	J-012949-07	7094	NM_006289	16753232	UCAAUCAGCUCAUCACUAU
TLN1	J-012949-08	7094	NM_006289	16753232	GAGAUGAGGAGUCUACUAU
ITGA5	J-008003-08	3678	NM_002205	56237028	GAACGAGUCAGAAUUUCGA
ITGA5	J-008003-09	3678	NM_002205	56237028	UCACAUCGCUCUCAACUUC
ITGA5	J-008003-10	3678	NM_002205	56237028	ACACGUUGCUGACUCCAUU
ITGA5	J-008003-11	3678	NM_002205	56237028	CAAACGCUCCCUCCCAUAU
CD44	J-009999-09	960	NM_001001392	48255942	GAUCAACAGUGGCAAUGGA
CD44	J-009999-08	960	NM_001001392	48255942	CGAAGAAGGUGUGGGCAGA
CD44	J-009999-07	960	NM_001001392	48255942	CAAGUGGACUCAACGGAGA

CD44	J-009999-06	960	NM_001001392	48255942	GAAUAUAACCUGCCGCUUU
ITGA3	J-004571-09	3675	NM_005501	6006010	CCAAGGAAACCUCUAUAUU
ITGA3	J-004571-10	3675	NM_005501	6006010	GCGCAAGGAGUGGGACUUA
ITGA3	J-004571-11	3675	NM_005501	6006010	GGAGUGGCCCUACGAAGUC
ITGA3	J-004571-12	3675	NM_005501	6006010	GUGUACAUCUAUCACAGUA
ANLN	J-006838-12	54443	NM_018685	31657093	ACGCAACACUUUUGAAUUA
ANLN	J-006838-11	54443	NM_018685	31657093	GAUCAAGCAUUAGCAGAAA
ANLN	J-006838-10	54443	NM_018685	31657093	GGCGAUGCCUCUUUGAAUA
ANLN	J-006838-09	54443	NM_018685	31657093	GCAAACAACUAGAAACCAA
AR	J-003400-05	367	NM_001011645	58535454	GAGCGUGGACUUUCCGGAA
AR	J-003400-06	367	NM_001011645	58535454	UCAAGGAACUCGAUCGUAU
AR	J-003400-07	367	NM_001011645	58535454	CGAGAGAGCUGCAUCAGUU
AR	J-003400-08	367	NM_001011645	58535454	CAGAAAUGAUUGCACUAUU
EPHA2	J-003116-09	1969	NM_004431	32967310	UGAAUGACAUGCCGAUCUA
EPHA2	J-003116-10	1969	NM_004431	32967310	GAAGUUCACUACCGAGAUC
EPHA2	J-003116-11	1969	NM_004431	32967310	CAAGUUCGCUGACAUCGUC
EPHA2	J-003116-12	1969	NM_004431	32967310	UCACACACCCGUAUGGCAA
FERMT2	J-012753-05	10979	NM_006832	29789005	GCCCAGGACUGUAUAGUAA
FERMT2	J-012753-06	10979	NM_006832	29789005	CUACAUAUUUCUCUCAACA
FERMT2	J-012753-07	10979	NM_006832	29789005	GAACUGAGUGUCCAUGUGA
FERMT2	J-012753-08	10979	NM_006832	29789005	AAUGAAAUCUGGCUUCGUU
LAMA4	J-011712-05	3910	NM_002290	9845494	GAUAUGAACUGAUAGUAGA
LAMA4	J-011712-06	3910	NM_002290	9845494	CCAGAAGACUCACUUAUAU
LAMA4	J-011712-07	3910	NM_002290	9845494	CCAGUGAGCUUCAGUAAAG
LAMA4	J-011712-08	3910	NM_002290	9845494	GAACCAGUAUGGAUGACUU
ITGB1	J-004506-05	3688	NM_033668	19743818	GUGCAGAGCCUUCAAUAAA
ITGB1	J-004506-06	3688	NM_033668	19743818	GGUAGAAAGUCGGGACAAA
ITGB1	J-004506-07	3688	NM_033668	19743818	UGAUAGAUCCAAUGGCUUA
ITGB1	J-004506-08	3688	NM_033668	19743818	GGGCAAACGUGUGAGAUGU
SRF	J-009800-07	6722	NM_003131	61743976	UGAGACAGGCCAUGUGUAU
SRF	J-009800-08	6722	NM_003131	61743976	GGACUGUGCUGAAGAGUAC
SRF	J-009800-09	6722	NM_003131	61743976	GCACCAAGAGUGAAUGAUC
SRF	J-009800-10	6722	NM_003131	61743976	GCACCAGUGUCUGCUAGUG
LAMC1	J-011714-05	3915	NM_002293	9845497	GCAAGCAGGUGUUGAGUUA
LAMC1	J-011714-06	3915	NM_002293	9845497	GAGAAGUGCUGGAUAUUUG
LAMC1	J-011714-07	3915	NM_002293	9845497	CAAAUUAGCCGUUUACAGA
LAMC1	J-011714-08	3915	NM_002293	9845497	GAUCAAGCCUUCGAGGAUA
LAMB2	J-013310-05	3913	NM_002292	9845495	GAGAUAGGCUUGCACUUAA
LAMB2	J-013310-06	3913	NM_002292	9845495	UGAAAGGGCUCGGCAGUUG
LAMB2	J-013310-07	3913	NM_002292	9845495	GUACCGAUAUUUCUCCUAU
LAMB2	J-013310-08	3913	NM_002292	9845495	CGACUGGGCCUACUGCUAA

ITGA1	J-008516-12	3672	NM_181501	31657141	CCUUCUACAUGUUGGACAA
ITGA1	J-008516-11	3672	NM_181501	31657141	CAGCUAUAACCGAGGAAAU
ITGA1	J-008516-10	3672	NM_181501	31657141	GGAUUUAAAUGGUGACGGU
ITGA1	J-008516-09	3672	NM_181501	31657141	AGACAAAUAUCACGAAGUU
MYOF	J-013584-12	26509	NM_133337	19718758	CGGCGGAUGCUGUCAAAUA
MYOF	J-013584-11	26509	NM_133337	19718758	GAUUGAGGGCCGACAGUUA
MYOF	J-013584-10	26509	NM_133337	19718758	GGCGAAGGCUGGUCCGAAA
MYOF	J-013584-09	26509	NM_133337	19718758	UUAUAGAAGACACGAGAUA
ITGA7	J-008004-09	3679	NM_002206	4504752	GAGGAGUACUCAGCUGUGA
ITGA7	J-008004-10	3679	NM_002206	4504752	GGCCCUCAAUAGCUACUUA
ITGA7	J-008004-11	3679	NM_002206	4504752	UAUGAUUGGUCGCUGCUUU
ITGA7	J-008004-12	3679	NM_002206	4504752	CAAGGCCAGUCGCUCAGAA
ILK	J-004499-05	3611	NM_001014794	62420872	GGGCACGGAUCAAUGUAAU
ILK	J-004499-06	3611	NM_001014794	62420872	CAAUAGCCGUAGUGUAAUG
ILK	J-004499-07	3611	NM_001014794	62420872	CGACCCAAAUUUGACAUGA
ILK	J-004499-08	3611	NM_001014794	62420872	GCACCAAUUUCGUCGUGGA
CAV3	J-011229-05	859	NM_001234	15451858	UCAAGGUGGUGCUGCGGAA
CAV3	J-011229-06	859	NM_001234	15451858	GCCCAGAUCGUCAAGGAUA
CAV3	J-011229-07	859	NM_001234	15451858	GGACAUAGUCAAGGUGGAU
CAV3	J-011229-08	859	NM_001234	15451858	UGCCAUGCAUUAAGAGCUA
HIF1A	J-004018-07	3091	NM_181054	31077210	GAACAAAUACAUGGGAUUA
HIF1A	J-004018-08	3091	NM_181054	31077210	AGAAUGAAGUGUACCCUAA
HIF1A	J-004018-09	3091	NM_181054	31077210	GAUGGAAGCACUAGACAAA
HIF1A	J-004018-10	3091	NM_181054	31077210	CAAGUAGCCUCUUUGACAA
PALLD	J-016891-08	23022	NM_016081	21361584	GAGUUUAUCUGGAGUGUAG
PALLD	J-016891-07	23022	NM_016081	21361584	CCAGUAACUUUCACAUGUA
PALLD	J-016891-06	23022	NM_016081	21361584	GUGCAACUGUCUUUAAUAU
PALLD	J-016891-05	23022	NM_016081	21361584	GAGCUAACAUCCAUAUUUA
LAMA2	J-011070-05	3908	NM_000426	28559087	GCAAUGACAUACUCGAUGA
LAMA2	J-011070-06	3908	NM_000426	28559087	GAAAGGAAUUUAUGACAGU
LAMA2	J-011070-07	3908	NM_000426	28559087	GCUCCUGUCUGAUAUGUAA
LAMA2	J-011070-08	3908	NM_000426	28559087	GCACUGGGCCACCGUCAUA
PARVA	J-005287-10	55742	NM_018222	19923815	UCGAGAAACUGGAGAGUGA
PARVA	J-005287-09	55742	NM_018222	19923815	GAUCCAAACUCACGCAGUG
PARVA	J-005287-08	55742	NM_018222	19923815	AGAAUGAGGUGCGAACAAU
PARVA	J-005287-07	55742	NM_018222	19923815	CCUGAAAUCUACACUACGA
YWHAG	J-008844-05	7532	NM_012479	21464100	GAUUAGGCCUGGCUCUUAA
YWHAG	J-008844-06	7532	NM_012479	21464100	GCGGCGAAGGCAACAAUUA
YWHAG	J-008844-07	7532	NM_012479	21464100	UGAAUGAGCCACUGUCGAA
YWHAG	J-008844-08	7532	NM_012479	21464100	GAGAAGGCCUACAGCGAAG
TWIST1	J-006434-07	7291	NM_000474	68160957	UGAGCAACAGCGAGGAAGA

TWIST1	J-006434-08	7291	NM_000474	68160957	GGAGUCCGCAGUCUUACGA
TWIST1	J-006434-09	7291	NM_000474	68160957	GCAAAUAGAUCCGGUGUCU
TWIST1	J-006434-10	7291	NM_000474	68160957	AAUCAGAGGAACUAUAAGA
PTRF	J-012807-12	284119	NM_012232	42734429	GCGAGAAACUGAAGACGUC
PTRF	J-012807-11	284119	NM_012232	42734429	CCAUCUCUACUAAGCGAAA
PTRF	J-012807-10	284119	NM_012232	42734429	CCUACUAGAAGGACGUGAA
PTRF	J-012807-09	284119	NM_012232	42734429	CGAGACAAGUUGCGCAAAU
CD59	J-004537-06	966	NM_000611	42716300	AAAAUGAGCUAACGUACUA
CD59	J-004537-07	966	NM_000611	42716300	GCAAGAAGGACCUGUGUAA
CD59	J-004537-08	966	NM_000611	42716300	GUGUGAGGCUGGCGCAUUA
CD59	J-004537-09	966	NM_000611	42716300	CAGAAUAGCUUGGUCCUUA
RAB34	J-009735-08	83871	NM_031934	31543536	GGGAAGACUUGCCUCAUUA
RAB34	J-009735-07	83871	NM_031934	31543536	GAUUUGAGGUGCUGGGCAU
RAB34	J-009735-06	83871	NM_031934	31543536	GUGAGAAUGUCCGAGAAUU
RAB34	J-009735-05	83871	NM_031934	31543536	GCAUCAACCUACUAUAGAG

Supplementary Figures S1-S5

Figure S1

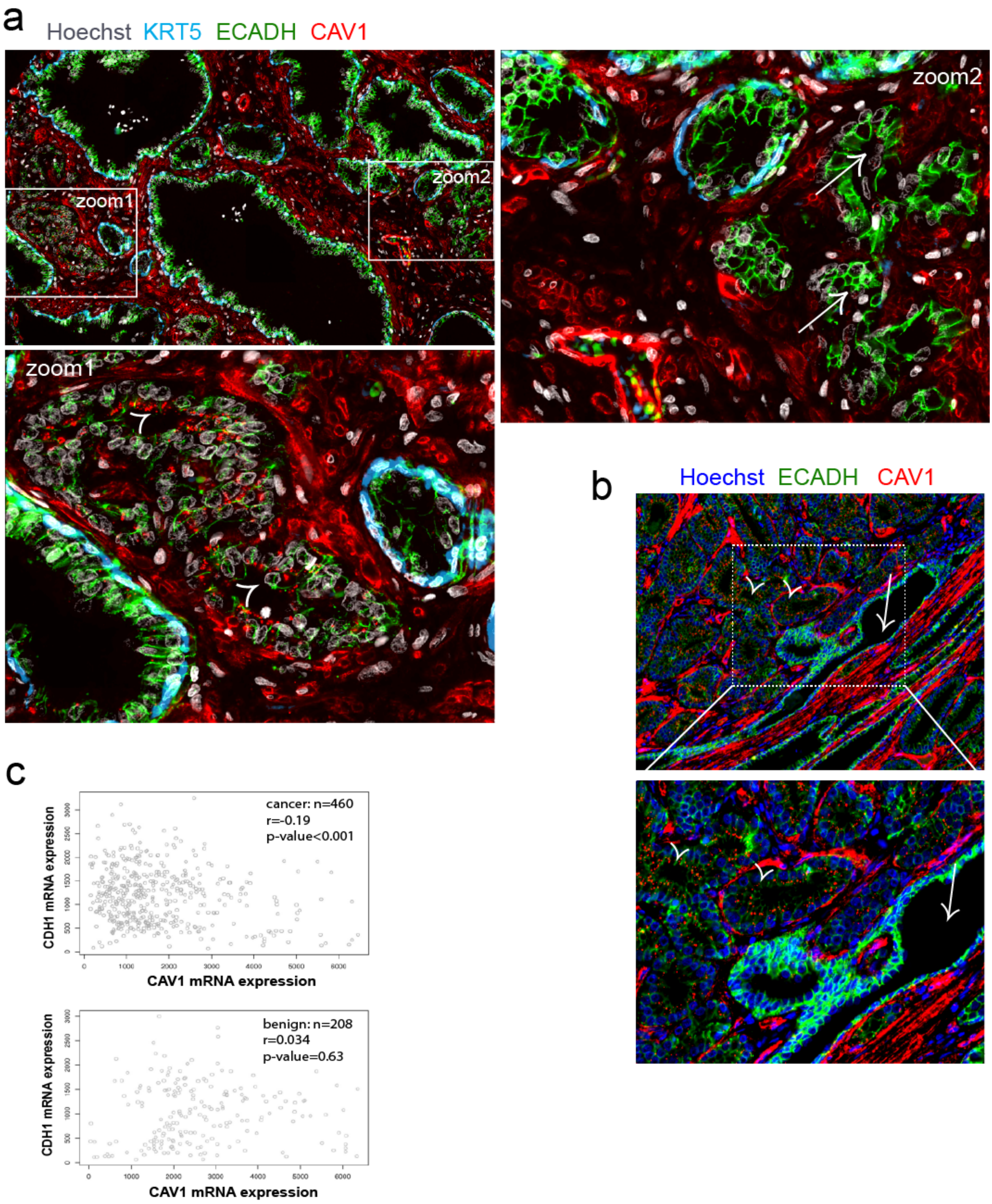

Figure S1. **CAV1 and E-cadherin show inverse association both at the inter-patient and intra-patient level in PCa**. (**a**) Mutually exclusive expression of CAV1 and E-cadherin (ECADH) in a PCa patient. The zoom1 area shows granular CAV1 expression with reduced ECADH expression (arrow-heads) as compared to the non-malignant KRT5-positive glands. The same patient has no CAV1 expression in the cancer glands of another region (zoom2), but shows strong ECADH expression (arrows). (**b**) An example of increased CAV1 expression and reduced ECADH expression in cancer glands (arrow-heads) adjacent to a non-malignant gland (arrow). (**c**) CAV1 and ECADH mRNA (CDH1) expressions show inverse correlation in PCa patients (upper image), while benign samples show no association (lower image). The mRNA expression data was from GeneSapiens Ltd.

Figure S2

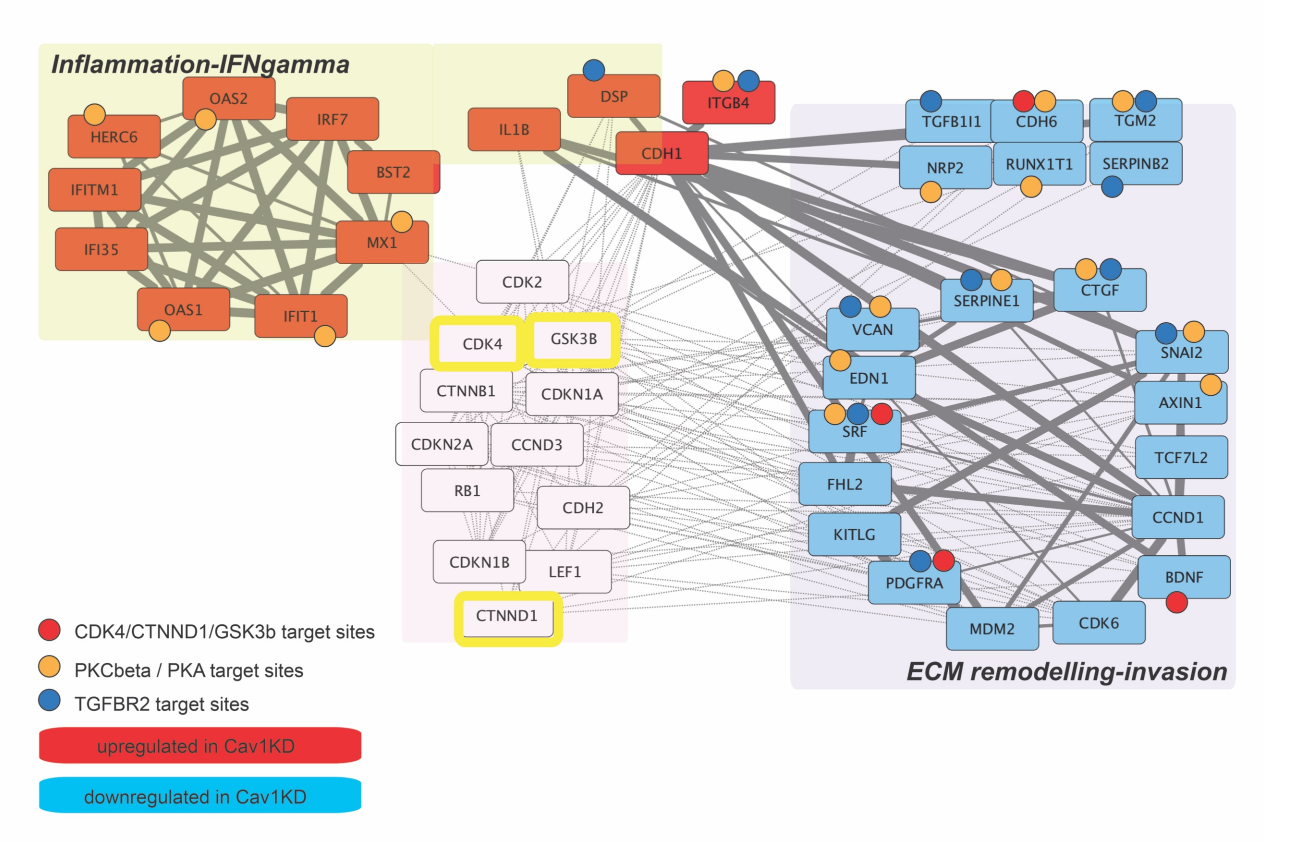

Figure S2. **Gene network emerging from genes significantly impacted by CAV1 knockdown.** Hits listed in Supplementary Table S4 were queried for relationships based on PPI, literature or curated genetic interactions, specifically filtering co-expression relationships to avoid redundancy.

Searches were performed on the STRING platform at moderate confidence (0.65 threshold), allowing for 5 additional interactors in both inner and outer shell (clear boxes and thin edges linking the two main modules). The resulting network is overlaid with functional grouping as annotated by IPA platform (coloured hue boxes), and the presence of phosphorylation acceptor sites predicted by the NetworKIN algorithm for the indicated kinase groups. Network modelling recapitulates the distribution in two distinct, functionally coherent modules of the differentially expressed genes. Yellow boxes indicate kinases, which are predicted to have substrates within the CAV1-associated clusters.

Figure S3

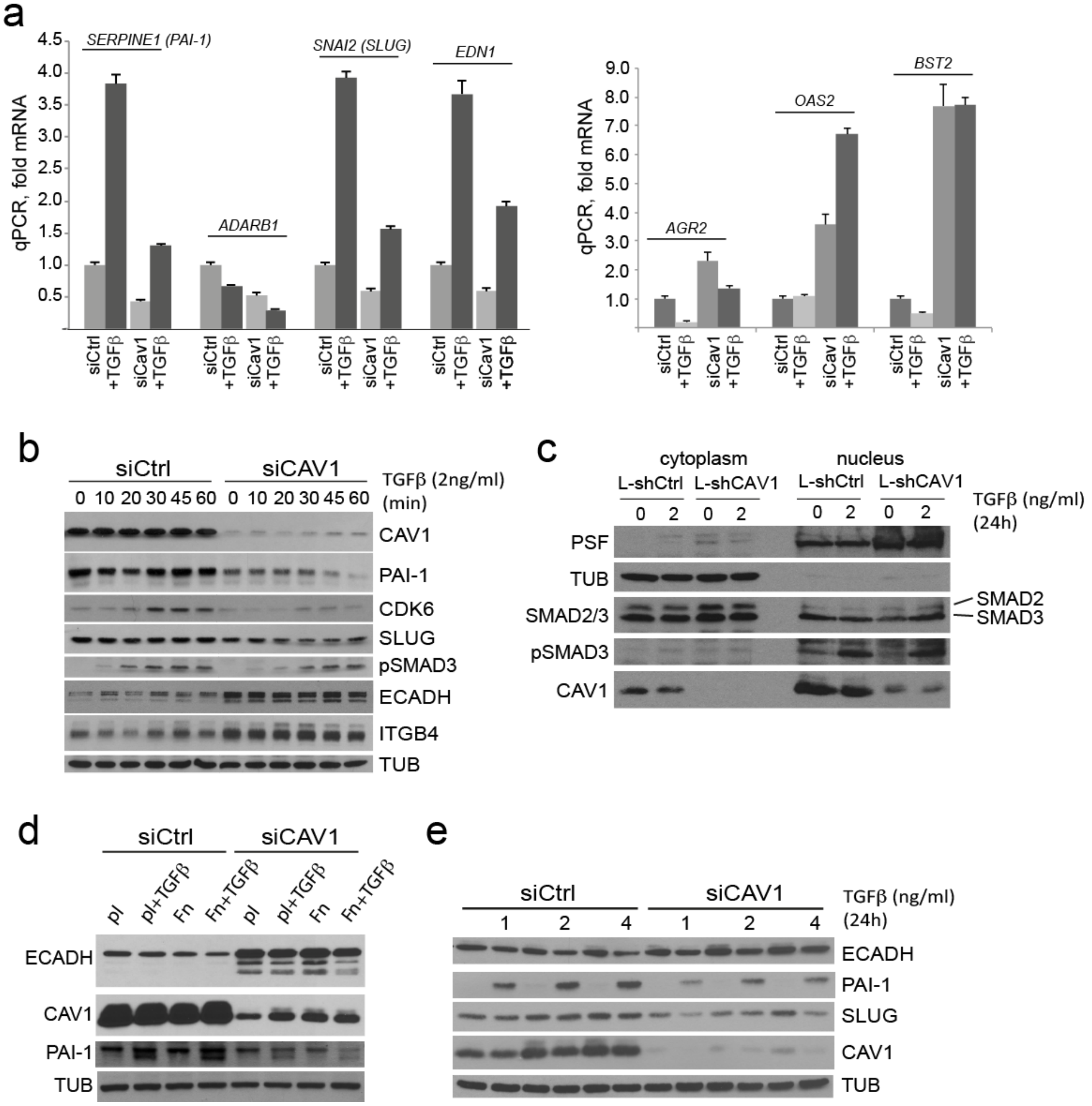

Figure S3. CAV1 regulates TGFβ target genes. (**a**) CAV1-silenced or control-silenced PC-3 cells were treated with TGFβ and analysed with qPCR for gene transcripts to validate selected targets of the microarray experiment. (**b**) Control siRNA or siRNA against CAV1 were used for transfecting PC-3 cells as indicated and lysates analysed with Western blotting for targets found in the microarray experiment. Phospho-SMAD3 was used as a positive control for TGFβ induction. (**c**) Cytoplasmic and nuclear lysates of PC-3 cells were immunoblotted for SMAD2/3 and pSMAD3 to analyse the

effect of CAV1 silencing on Smad2/3 localization. PSF was used as a control for nuclear fractions (TUB = Tubulin; PSF = PTB-associated splicing factor). (**d**) DU145 cells treated as indicated and analysed with Western blotting. See that the samples used here were the same as in Figure 3a. (**e**) PNT2 prostate cells (immortalized benign) were silenced and treated as indicated and blotted for E-cadherin (ECADH), SLUG, and PAI-1.

Figure S4

a

	Plate	z-prime	robust z-prime
PC-3	plate 1	0.887	0.885
	plate 2	0.818	0.880
	plate 3	0.878	0.874
DU145	plate 1	0.941	0.946
	plate 2	0.706	0.630
	plate 3	0.671	0.666
PNT2	plate 1	0.738	0.725
	plate 2	0.615	0.723
	plate 3	0.599	0.565

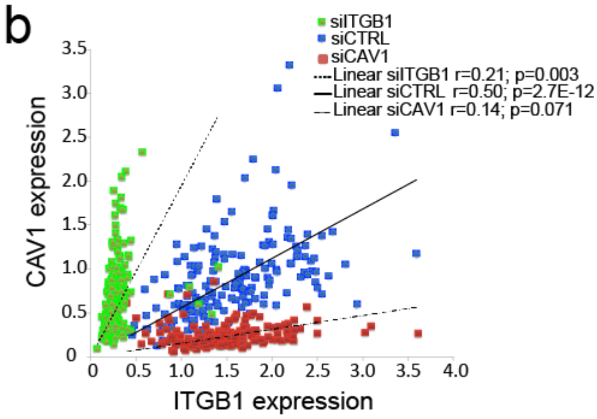

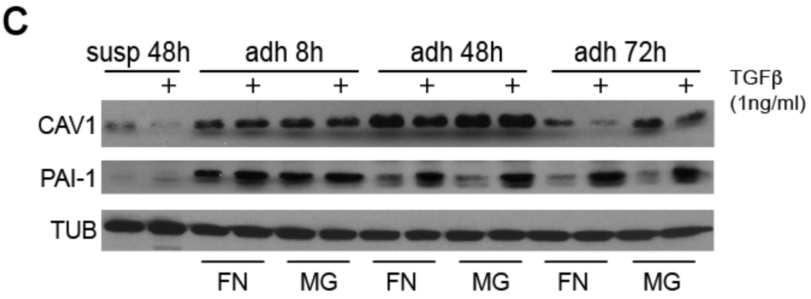

Figure S4. **CAV1 expression regulation by beta1 integrins**. (**a**) RNAi screen z-prime and robust z-prime values for each cell line and replicate plate. Z-prime is a measure commonly used in High Content Screening to quantify the distance between the positive and negative controls as well as the dynamic range of the controls. Z-prime values higher than 0.5 are considered as GOOD. (**b**) Scatter plot illustration of CAV1 and ITGB1 protein expression at cellular level upon silencing with indicated siRNAs in Du145 cells. Each dot represents one segmented cell in the function of ITGB1 and CAV1 immunofluorescent staining intensities. The example result is from a different replicate plate than the siCtrl result in Fig. 5C. (**c**) Western blots of lysates from PC-3 cells adherent to

fibronectin- (FN) or Matrigel (MG) coated plates with or without TGFβ for indicated times. Alternatively, cells were grown in suspension (susp) for 48h. Cells adherent for 72h reached confluence.

Figure S5

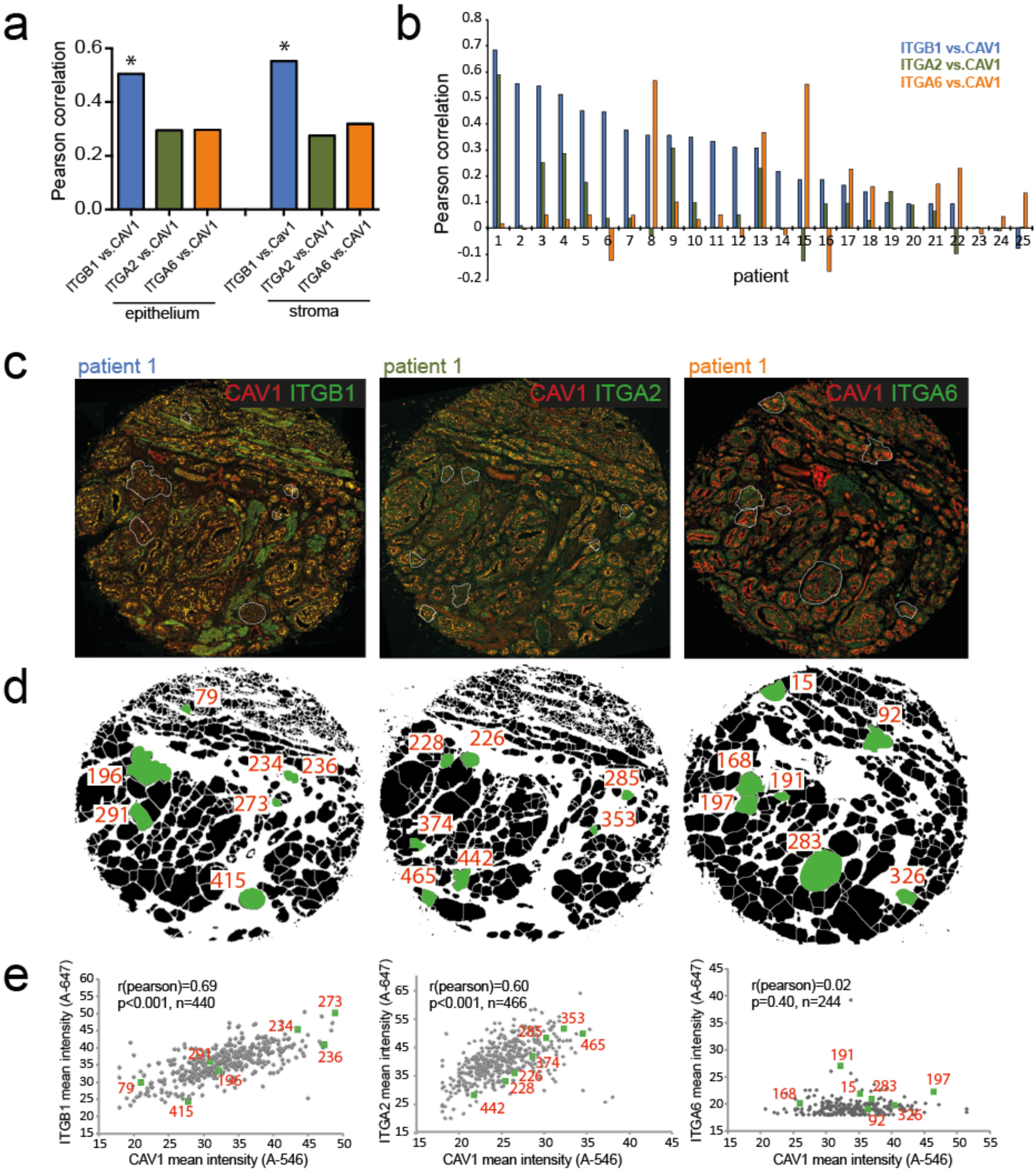

Figure S5. **CAV1 is co-expressed with beta1 integrins in clinical prostate cancer**. **(a-b)** Co-expression of CAV1 with ITGB1, ITGA2, and ITGA6 across different PCa patients' epithelium or

stroma (A) (n=25, *p<0.01, two-tailed Student's *t* test) or within each patients' epithelium only (b). (**c-e**) Intra-patient co-expression of CAV1 with integrins ITGB1, ITGA2, and ITGA6 within segmented epithelial objects in patient 1. Each dot in (e) represents one epithelial object as defined by the segmentation mask based on pan-cytokeratin positivity (d, black areas as masks). Example objects are highlighted with numbers and with green colour. The results demonstrate high co-expression of CAV1 and ITGB1 across the patients, but also point out that different ITGB1-binding alpha-integrins may also be co-expressed with CAV1, however, with some variation between patients.

SUPPLEMENTARY REFERENCES

1. Kilpinen, S., *et al.* Systematic bioinformatic analysis of expression levels of 17,330 human genes across 9,783 samples from 175 types of healthy and pathological tissues. Genome biology **9**, R139 (2008).
2. Zavadil, J., *et al.* Genetic programs of epithelial cell plasticity directed by transforming growth factor-beta. Proc Natl Acad Sci U S A **98**, 6686-6691 (2001).
3. Levy, L. & Hill, C.S. Smad4 dependency defines two classes of transforming growth factor {beta} (TGF-{beta}) target genes and distinguishes TGF-{beta}-induced epithelial-mesenchymal transition from its antiproliferative and migratory responses. Mol Cell Biol **25**, 8108-8125 (2005).
4. Kang, Y., Chen, C.R. & Massague, J. A self-enabling TGFbeta response coupled to stress signaling: Smad engages stress response factor ATF3 for Id1 repression in epithelial cells. Mol Cell **11**, 915-926 (2003).
5. Gaspar, N.J., *et al.* Inhibition of transforming growth factor beta signaling reduces pancreatic adenocarcinoma growth and invasiveness. Mol Pharmacol **72**, 152-161 (2007).
6. Qin, H., *et al.* An integrative ChIP-chip and gene expression profiling to model SMAD regulatory modules. BMC Syst Biol **3**, 73 (2009).
7. Sancisi, V., *et al.* Cadherin 6 is a new RUNX2 target in TGF-beta signalling pathway. PLoS One **8**, e75489 (2013).
8. Kahari, V.M., Larjava, H. & Uitto, J. Differential regulation of extracellular matrix proteoglycan (PG) gene expression. Transforming growth factor-beta 1 up-regulates biglycan (PGI), and versican (large fibroblast PG) but down-regulates decorin (PGII) mRNA levels in human fibroblasts in culture. J Biol Chem **266**, 10608-10615 (1991).
9. Yeung, T.L., *et al.* TGF-beta modulates ovarian cancer invasion by upregulating CAF-derived versican in the tumor microenvironment. Cancer Res **73**, 5016-5028 (2013).
10. Saito, A., *et al.* An integrated expression profiling reveals target genes of TGF-beta and TNF-alpha possibly mediated by microRNAs in lung cancer cells. PLoS One **8**, e56587 (2013).

11. Sartor, M.A., *et al.* ConceptGen: a gene set enrichment and gene set relation mapping tool. Bioinformatics **26**, 456-463 (2010).
12. Karlsson, G., *et al.* Gene expression profiling demonstrates that TGF-beta1 signals exclusively through receptor complexes involving Alk5 and identifies targets of TGF-beta signaling. Physiol Genomics **21**, 396-403 (2005).
13. Sandbo, N., Kregel, S., Taurin, S., Bhorade, S. & Dulin, N.O. Critical role of serum response factor in pulmonary myofibroblast differentiation induced by TGF-beta. Am J Respir Cell Mol Biol **41**, 332-338 (2009).
14. Wang, X., *et al.* Transforming growth factor-beta1-induced transcript 1 protein, a novel marker for smooth muscle contractile phenotype, is regulated by serum response factor/myocardin protein. J Biol Chem **286**, 41589-41599 (2011).
15. Kim, D.J., *et al.* Lysyl oxidase like 4, a novel target gene of TGF-beta1 signaling, can negatively regulate TGF-beta1-induced cell motility in PLC/PRF/5 hepatoma cells. Biochem Biophys Res Commun **373**, 521-527 (2008).
16. Keshamouni, V.G., *et al.* Temporal quantitative proteomics by iTRAQ 2D-LC-MS/MS and corresponding mRNA expression analysis identify post-transcriptional modulation of actin-cytoskeleton regulators during TGF-beta-Induced epithelial-mesenchymal transition. J Proteome Res **8**, 35-47 (2009).
17. Lindemann, R.K., Nordheim, A. & Dittmer, J. Interfering with TGFbeta-induced Smad3 nuclear accumulation differentially affects TGFbeta-dependent gene expression. Mol Cancer **2**, 20 (2003).
18. Miettinen, P.J., Ebner, R., Lopez, A.R. & Derynck, R. TGF-beta induced transdifferentiation of mammary epithelial cells to mesenchymal cells: involvement of type I receptors. J Cell Biol **127**, 2021-2036 (1994).
19. Yan, M., *et al.* 15-Hydroxyprostaglandin dehydrogenase, a COX-2 oncogene antagonist, is a TGF-beta-induced suppressor of human gastrointestinal cancers. Proc Natl Acad Sci U S A **101**, 17468-17473 (2004).
20. Ruau, D., Ju, X.S. & Zenke, M. Genomics of TGF-beta1 signaling in stem cell commitment and dendritic cell development. Cell Immunol **244**, 116-120 (2006).
21. Shin, J.A., *et al.* Transforming growth factor-beta induces epithelial to mesenchymal transition and suppresses the proliferation and transdifferentiation of cultured human pancreatic duct cells. J Cell Biochem **112**, 179-188 (2011).

Original full scans of Western blots

Figure 3A

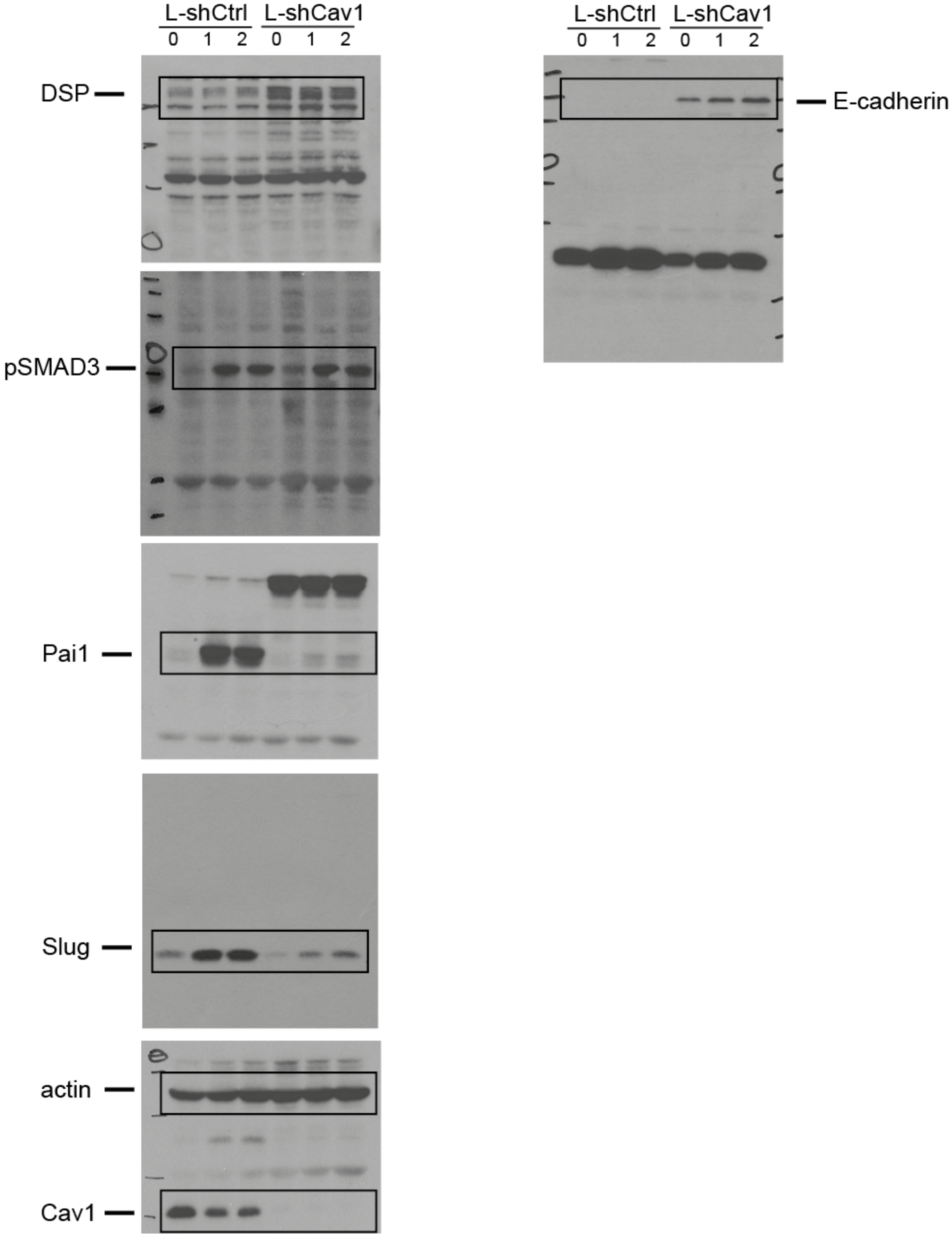

Figure 3B

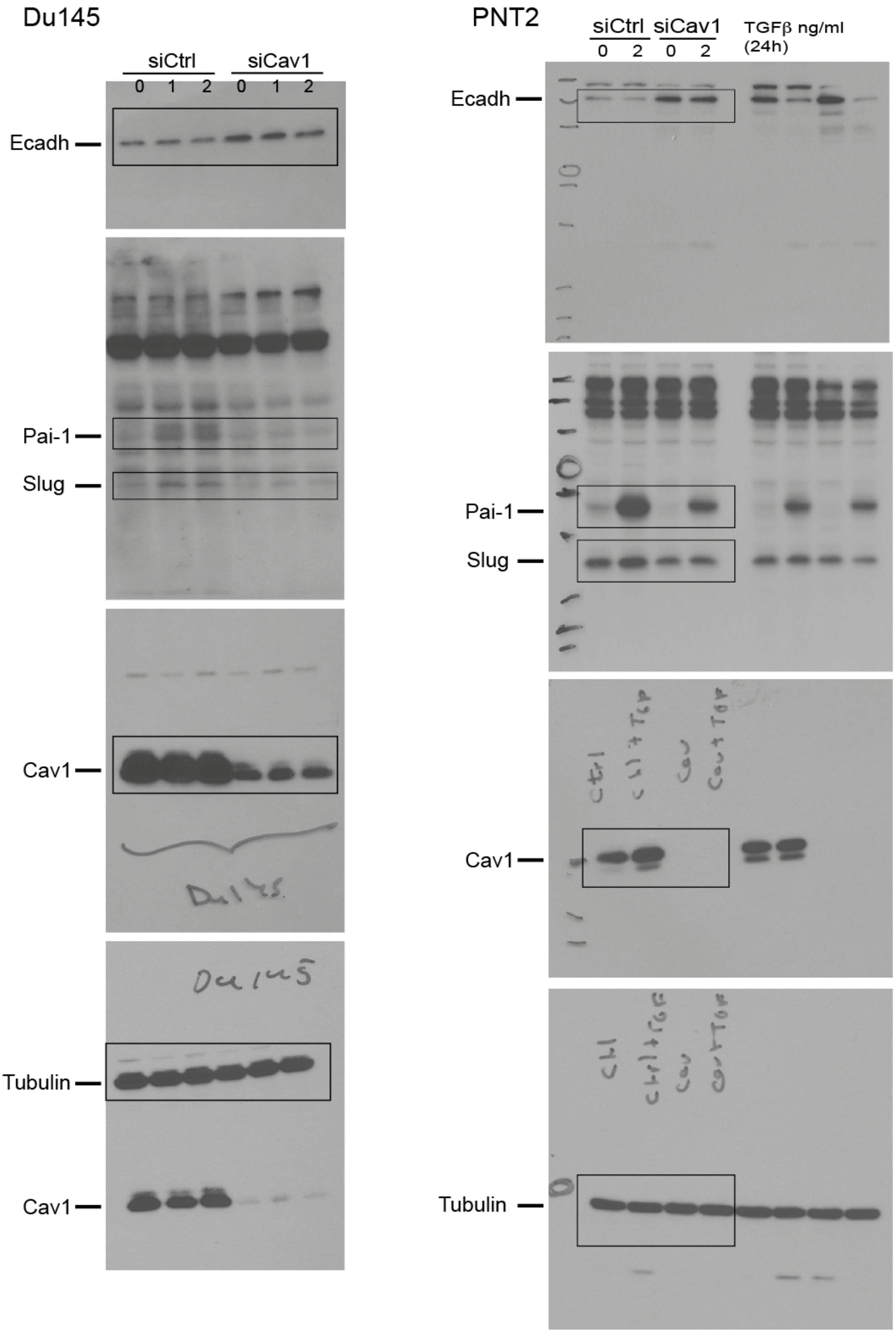
Du145
siCtrl
siCav1
0 1 2 0 1 2
Ecadh
Pai-1
Slug
Cav1
Tubulin
Cav1
PNT2
siCtrl
siCav1
0 2 0 2
TGFβ ng/ml
(24h)
Ecadh
Pai-1
Slug
Cav1
Tubulin

Figure 3C

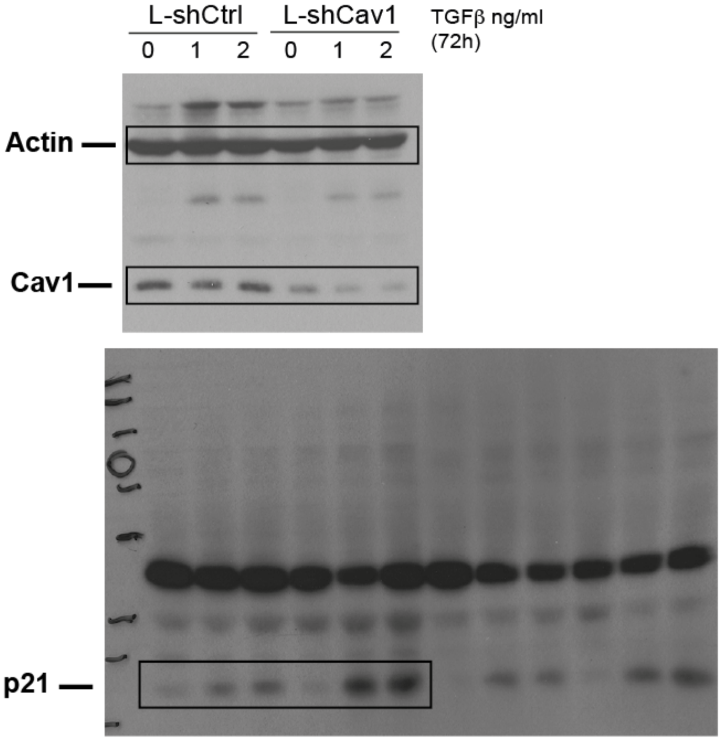

Figure 3E

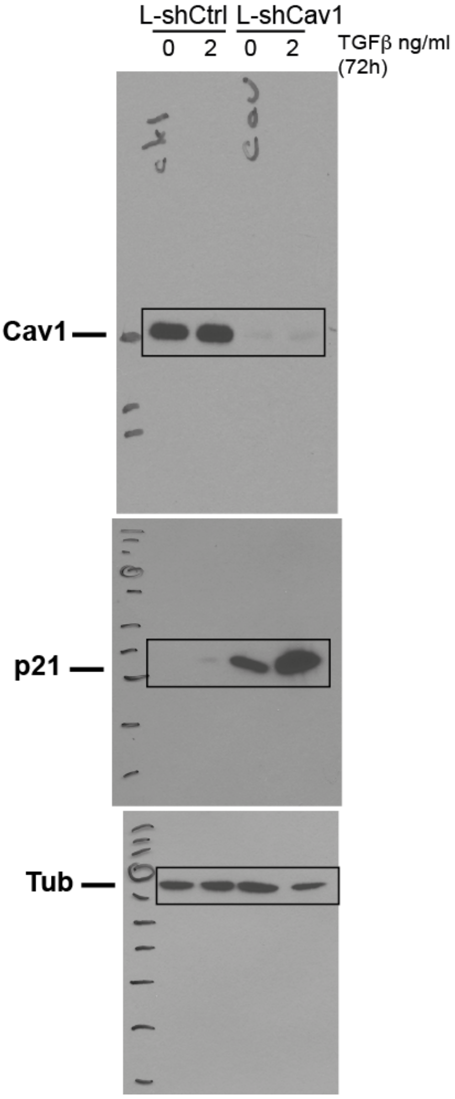

Figure 3D

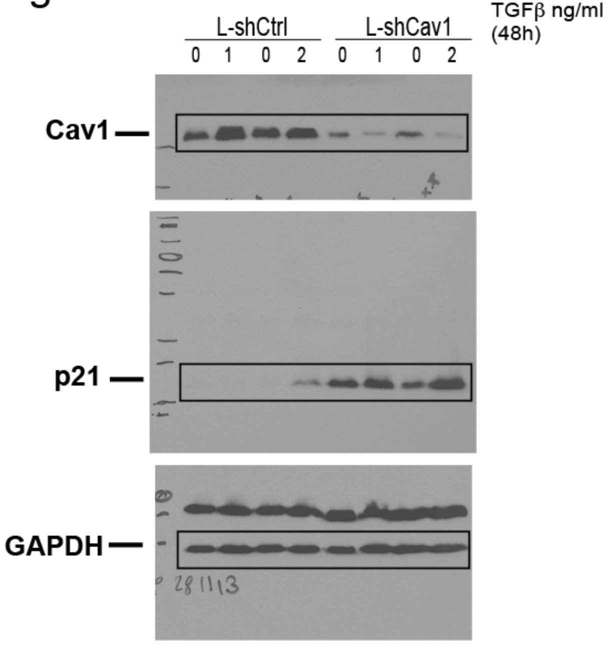

Figure 4B

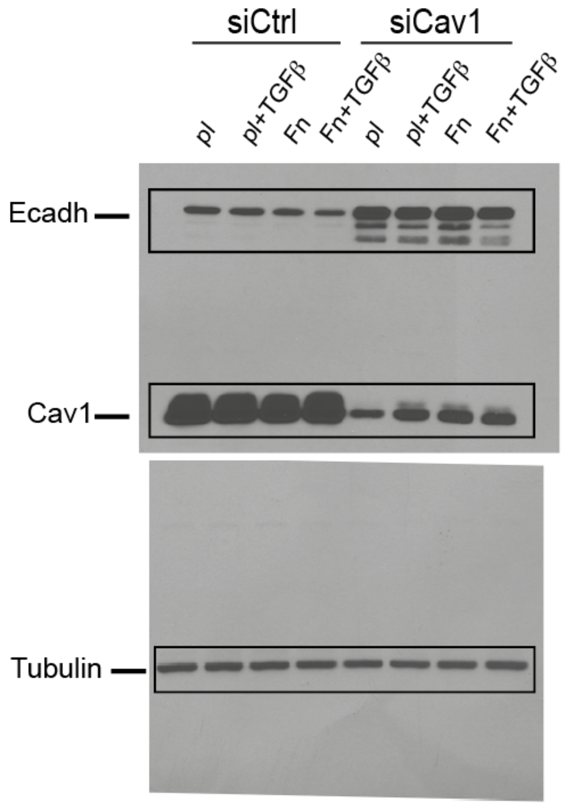

Figure 4D

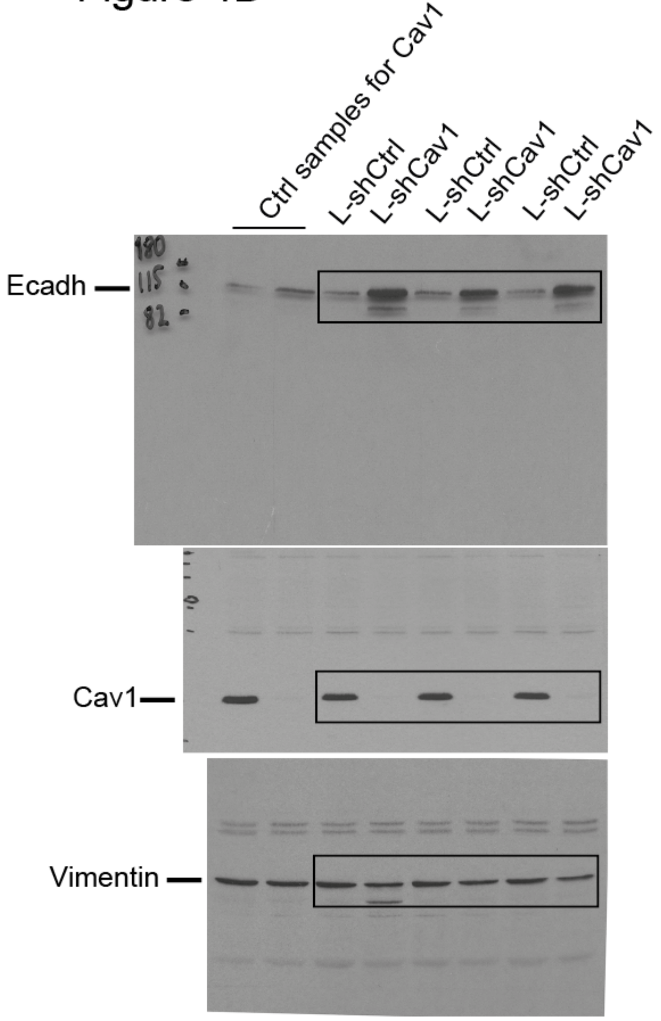

Figure 5E

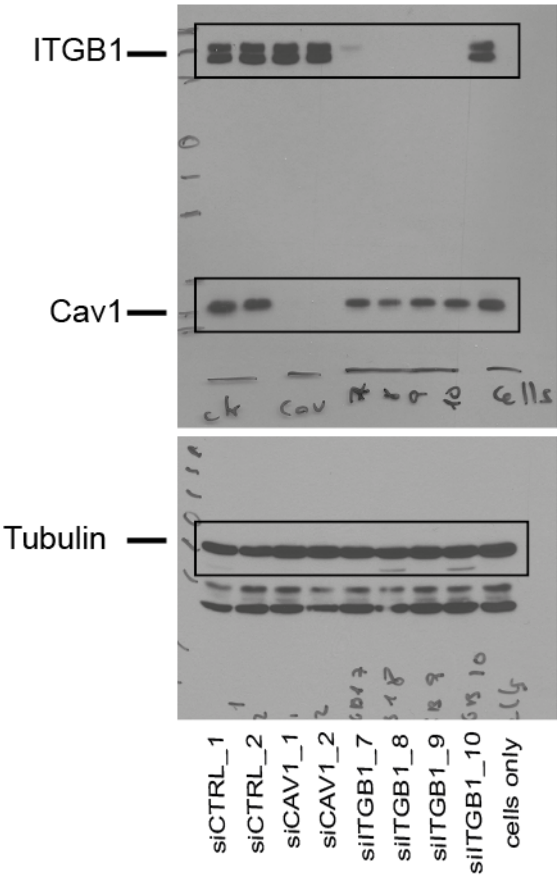
ITGB1
Cav1
Tubulin
siCTRL_1
siCTRL_2
siCAV1_1
siCAV1_2
siITGB1_7
siITGB1_8
siITGB1_9
siITGB1_10
cells only

Supplementary
Figure S3B

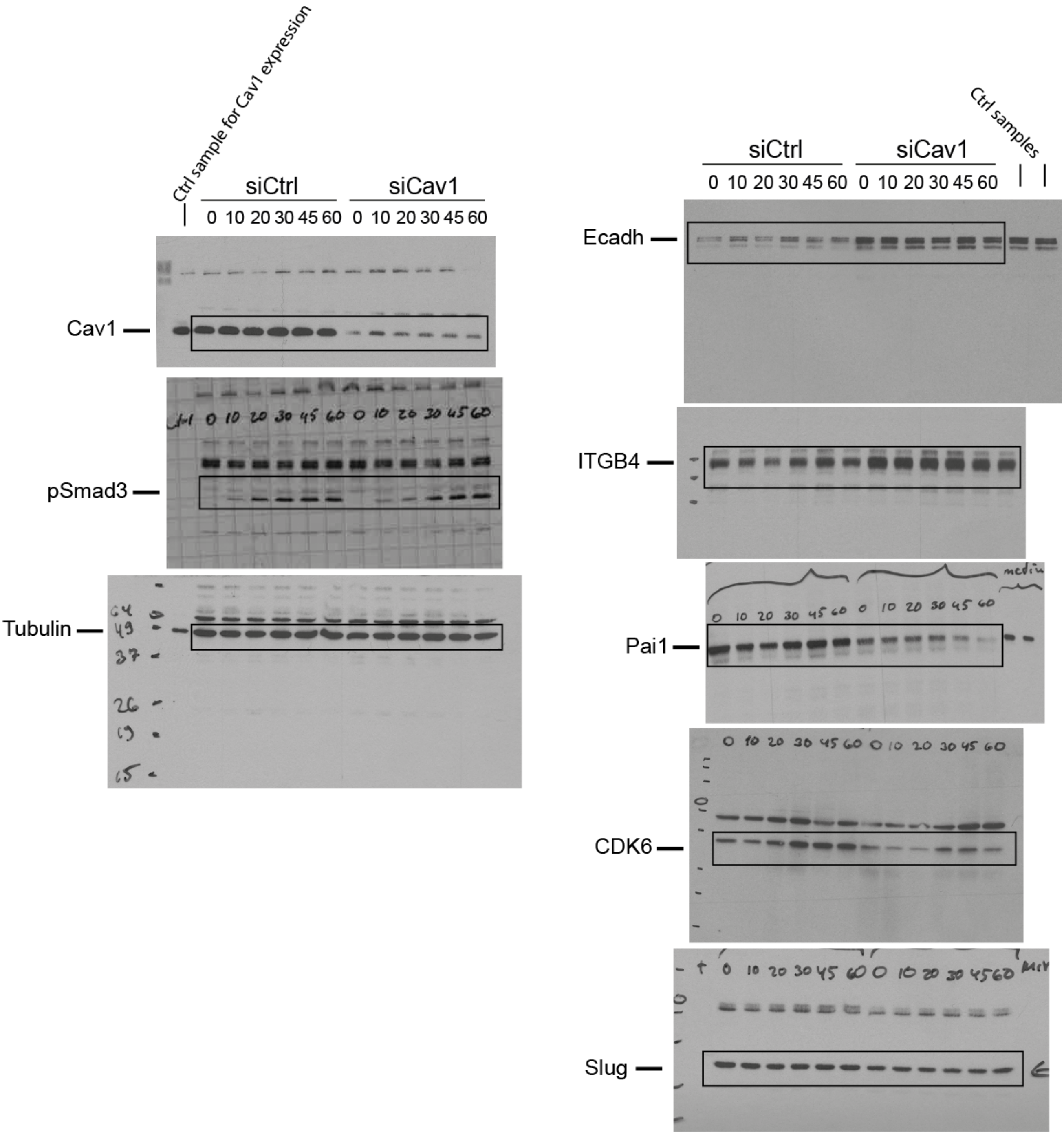

Supplementary Figure S3C

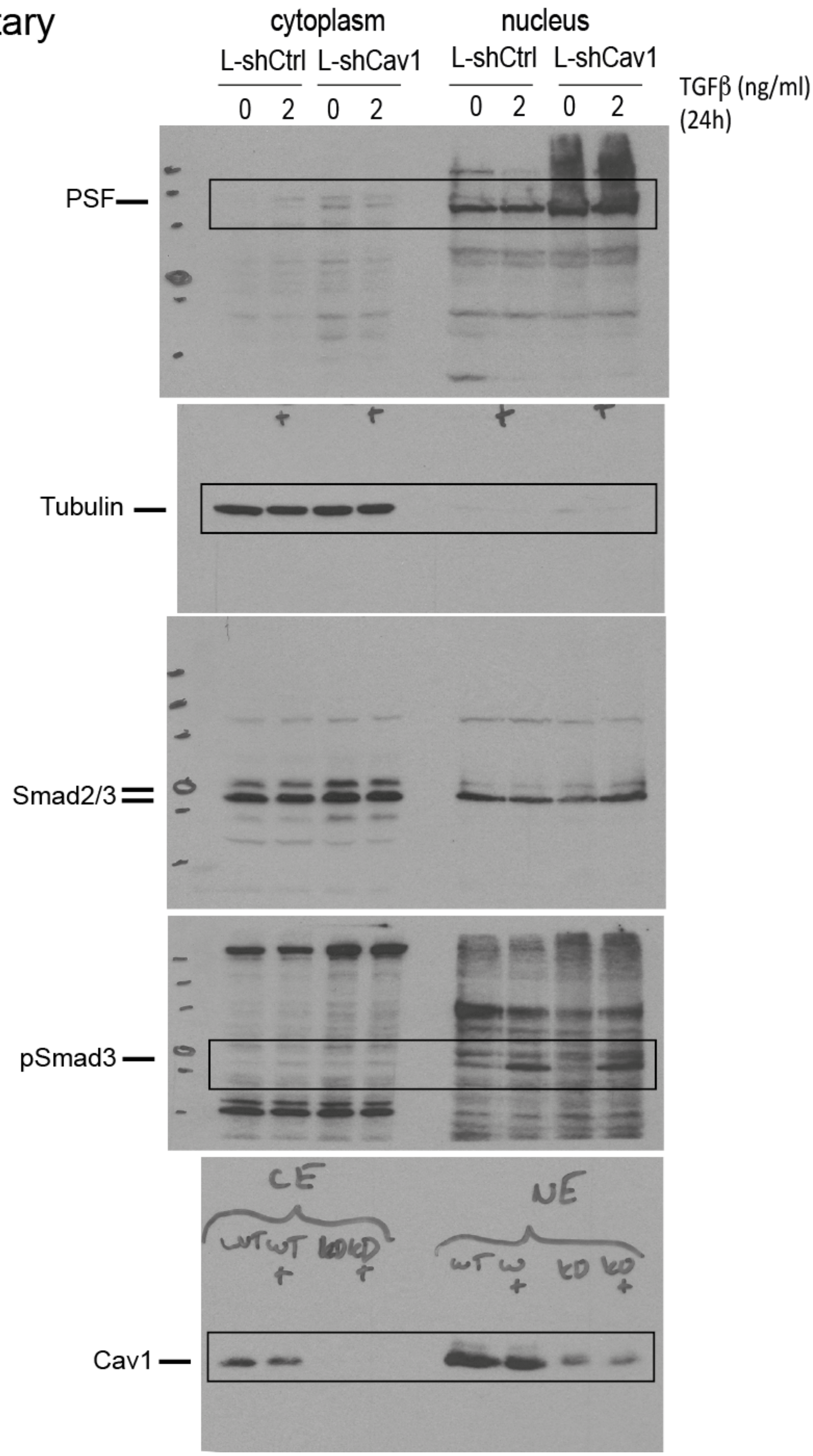

Supplementary Figure S3D

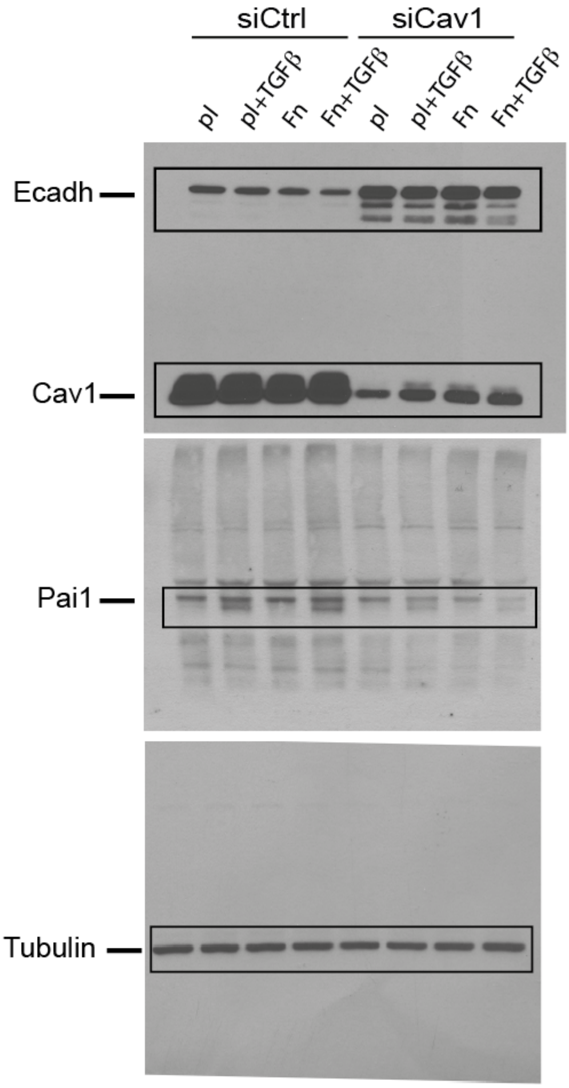

Supplementary Figure S3E

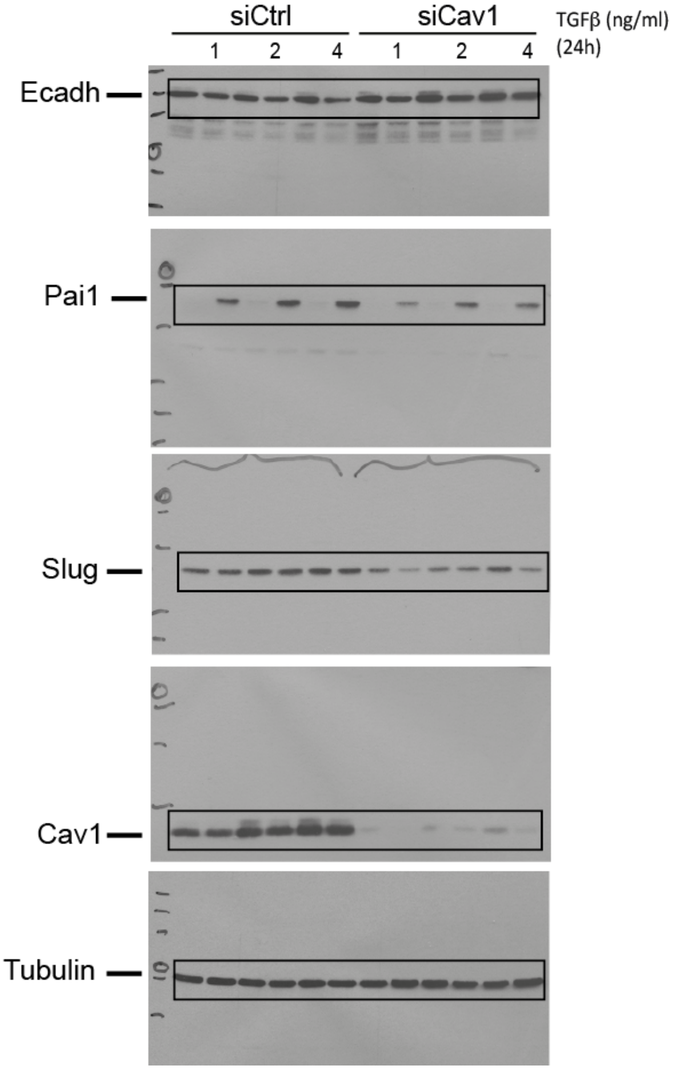

Supplementary Figure S4C

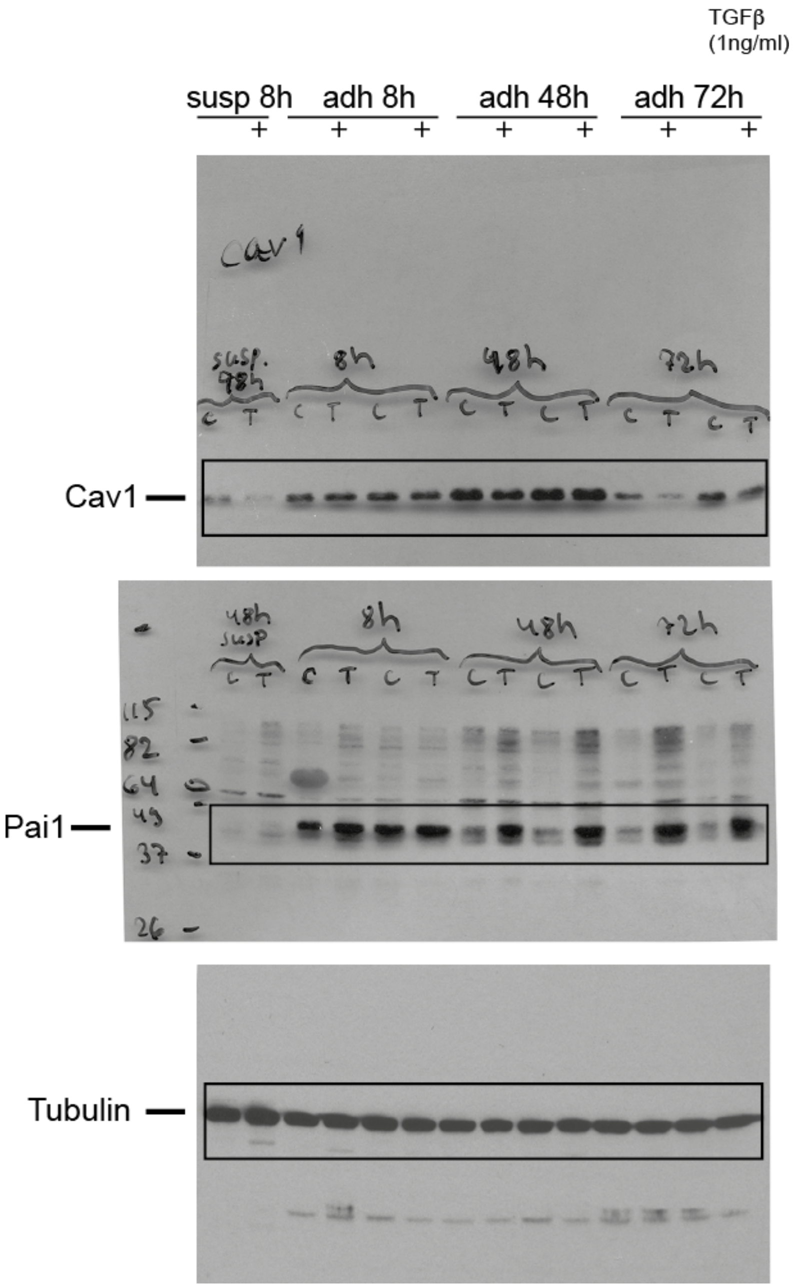

Supplement: Supplementary file 1 — Supplementary information [file 41598_2018_20161_MOESM1_ESM.pdf]
